# Effects of Nutrients, Temperature and Their Interactions on Spring Phytoplankton Community Succession in Lake Taihu, China

**DOI:** 10.1371/journal.pone.0113960

**Published:** 2014-12-02

**Authors:** Jianming Deng, Boqiang Qin, Hans W. Paerl, Yunlin Zhang, Pan Wu, Jianrong Ma, Yuwei Chen

**Affiliations:** 1 State Key Laboratory of Lake Science and Environment, Nanjing Institute of Geography and Limnology, Academy of Sciences, 73 East Beijing Road, Nanjing, 210008, P. R. China; 2 University of Chinese Academy of Sciences, Beijing, 100049, P.R. China; 3 Institute of Marine Sciences, University of North Carolina at Chapel Hill, Morehead City, North Carolina, 28557, United States of America; CSIR- National institute of oceanography, India

## Abstract

We examined the potential effects of environmental variables, and their interaction, on phytoplankton community succession in spring using long-term data from 1992 to 2012 in Lake Taihu, China. Laboratory experiments were additionally performed to test the sensitivity of the phytoplankton community to nutrient concentrations and temperature. A phytoplankton community structure analysis from 1992 to 2012 showed that *Cryptomonas* (Cryptophyta) was the dominant genus in spring during the early 1990s. Dominance then shifted to *Ulothrix* (Chlorophyta) in 1996 and 1997. However, *Cryptomonas* again dominated in 1999, 2000, and 2002, with *Ulothrix* regaining dominance from 2003 to 2006. The bloom-forming cyanobacterial genus *Microcystis* dominated in 1995, 2001 and 2007–2012. The results of ordinations indicated that the nutrient concentration (as indicated by the trophic state index) was the most important factor affecting phytoplankton community succession during the past two decades. In the laboratory experiments, shifts in dominance among phytoplankton taxa occurred in all nutrient addition treatments. Results of both long term monitoring and experiment indicated that nutrients exert a stronger control than water temperature on phytoplankton communities during spring. Interactive effect of nutrients and water temperature was the next principal factor. Overall, phytoplankton community composition was mediated by nutrients concentrations, but this effect was strongly enhanced by elevated water temperatures.

## Introduction

Phytoplankton community structure has been the subject of intense study for many decades. In general, increased nutrient loads are considered to be the driver of cyanobacterial dominance and blooms in lakes [1]. However, it has also been shown that dominance shifts from cyanobacteria to chlorophytes under excessive nutrient loading (i.e., hypertrophic) conditions [2] because in temperate lakes, chlorophytes are characterized by high growth and loss rates and they have a high demand for nutrients, whereas cyanobacteria have lower growth and loss rates and hence a lower demand for nutrients [3]. Based on previous works in shallow freshwater lakes, it is apparent that climate change may also cause qualitative changes in phytoplankton community dynamics, shown as changes in phytoplankton species composition or changes in the seasonal succession of phytoplankton groups [4]. Many studies have reported that rising temperatures enhance cyanobacterial biomass and dominance along a range of latitudes [5]–[7]. In addition, other aspects of climate change, including increases in rainfall and nutrient runoff are also intensifying the symptoms of eutrophication through the enhanced nutrient loading to lakes due to rainfall [8]–[10].

It was reported that nutrients are the more important predictor of cyanobacterial biovolume compared to water temperature as lakes become more eutrophic [11]. However, there is currently a critical knowledge gap in how eutrophication and climate variables individually and interactively impact the dynamics of marine ecosystems [12]. Being able to distinguish the individual and cumulative effects of physical, chemical and biotic controls of phytoplankton productivity and composition is key to understanding, predicting, and ultimately managing eutrophication [13].

Lake Taihu is the third largest freshwater lake in China. Previous studies on Lake Taihu have found that water temperature or accumulated water temperature was the principal force driving *Microcystis* blooms [14], [15]. Other studies have concluded that climatic variables rather than nutrients are crucial in predicting cyanobacterial bloom events because nutrients are present in sufficiently high quantities to sustain the formation of cyanobacterial blooms [16], [17]. It has also been reported that the spring season in the Lake Taihu region has become warmer [18], and as a result, the initiation time for cyanobacterial blooms has advanced further from summer into spring [16]. However, all the aforementioned studies on Lake Taihu have largely focused on bloom events or the harmful (toxic, food web disrupting, anoxia generating) genus *Microcystis*. This may lead to a bias in studying the effects of global change, eutrophication and their interactions on lake ecosystems, because specific phytoplankton taxa will response differently to nutrient enrichment and increasing water temperatures [19]–[21]. For instance, Thackeray et al. [22] demonstrated that nutrients play a more important role than water temperature when considered at the phytoplankton community level.

Knowledge of the mechanisms by which nutrients, temperature and their interactions affect the phytoplankton community succession in the subtropical shallow lakes remains limited. To address this information gap, we examined long-term monitoring data and conducted laboratory experiments in order to 1) assess the relative importance of temperature, nutrient concentrations and their interactions in driving phytoplankton community dynamics in spring in Lake Taihu, and 2) test whether conclusions based on the community level might be different from those based on single species.

## Materials and Methods

### Ethics Statement

No permits were required for the field studies, because the location was not privately-owned or protected, and the field studies did not involve endangered or protected species.

### Study site

**Figure 1:**
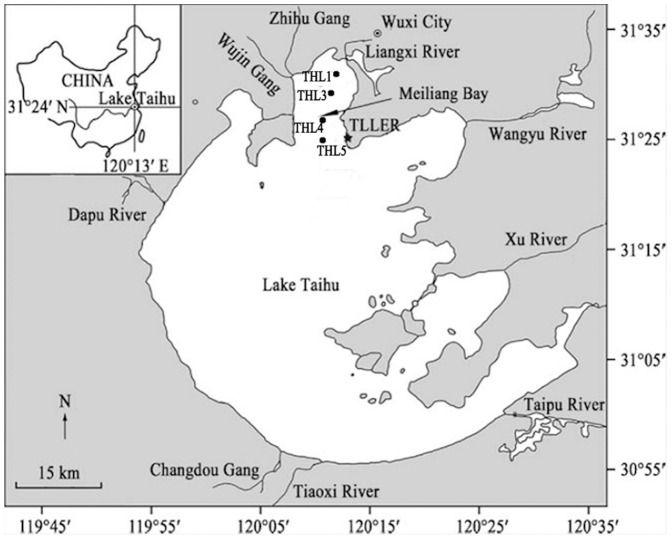
Location of Lake Taihu in China and the sampling sites. Map was redrawn from [26]. Phytoplankton biovolume together with water quality were monitored monthly at THL1#, THL3#, THL4# and THL5#, generally in the middle of each month.

Lake Taihu is a shallow, subtropical lake situated in the Changjiang (Yangtze) Delta (Figure 1). The lake is a polymictic [23], and as a result, differences in water temperature between the surface and the bottom are generally less than 1°C [24]. In 1960s, Lake Taihu was mesotrophic; however, by 1981 water quality had deteriorated. Currently, Lake Taihu is eutrophic [23]. Since the 1980's, rapid economic development in the Taihu basin has resulted in increasing levels of pollutants being discharged to tributaries emptying into the lake. As a result, rapid deterioration of water quality has occurred, accelerating eutrophication and increasing the frequency and intensity of cyanobacterial blooms (*Microcystis* spp.) [25].

Meiliang Bay is one of the lake's most eutrophic bays, located in the northern part of Lake Taihu. The blooms there are more intense than in most other regions of the lake [26]. The bay is also an intensively monitored region of the lake. Hence, Meiliang Bay was selected as our study area.

### Physicochemical variables

Four sampling sites (THL1#, THL3#, THL4# and THL5#, see Figure 1) were selected because they cover major sections of Meiliang Bay. Monthly sampling has been conducted at these sites since 1992. Surface water temperature (WT, °C) was measured with a mercury thermometer at 0.5 m below the water surface at the sampling sites. Integrated water samples were taken using a 2 m long, 10 cm diameter plastic tube. Physicochemical variables, including Secchi depth (SD, m), conductivity (Cond, µS·cm^−1^), chemical oxygen demand (COD, mg·L^−1^) and nutrients concentrations were analyzed following Chinese standard methods [27]. Specifically, total nitrogen (TN) and total phosphorus (TP) concentrations were determined using a combined persulfate digestion followed by spectrophotometric analysis as for soluble reactive phosphorus and nitrate. NH_4_^+^ concentrations were measured by the indophenol blue method, and NO_3_^−^ and NO_2_^−^ concentrations were analyzed by the cadmium reduction method [27], [28]. COD was measured by titration with acidic potassium permanganate. SD was determined by the classical procedure using a Secchi disk (diameter 0.3 m). Eight major ions (K^+^, Na^+^, Ca^2+^, Mg^2+^, Cl^−^, SO_4_^2−^, Si and F^−^) were measured by ion chromatography. Chlorophyll *a* (Chl *a*, µg·L^−1^) concentrations were determined spectrophotometrically after extraction in 90% hot ethanol [29]. Cumulative water temperature (CWT, °C) was calculated by summing each month's monitored surface water temperature in the spring season (March, April and May). The mean values of physical and chemical variables during the spring season were used in our analysis.

A tropic state index (TSI) was used to evaluate the trophic state of Lake Taihu. We used functions fitted according to original Table 6–11 in Wang and Dou [30] to calculate the TSI during our study. The TSI was calculated based on Chl-*a*, TN, TP, COD and SD (Equation 1). TSI<40 indicates an oligotrophic, 40≤TSI<80 a mesotrophic, and TSI≥80 a eutrophic state. The index for each month in the spring seasons from 1992 to 2012 was calculated, and then the mean index was calculated as a proxy of trophic state for the spring season of each year.
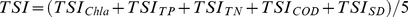
(1)Where 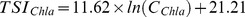


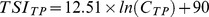


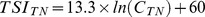


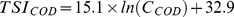


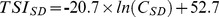



### Phytoplankton community dynamics

One liter of a vertically integrated sample was collected at each site for phytoplankton species identification and enumeration. Phytoplankton samples were fixed with Lugol's iodine solution and sedimented for 48 h prior to microscopic enumeration at 512× magnification. Phytoplankton species were identified according to Hu et al. [31] and Hu and Wei [32]. The phytoplankton community was identified to the genus level (whenever possible). Algal biovolumes were calculated from cell numbers and cell size measurements. Conversions to biomass assumed that 1 mm^3^ of volume was equivalent to 1 mg of fresh-weight biomass [33]. Phytoplankton biovolume data were not collected during 2004.

### Experimental design

**Figure 2:**
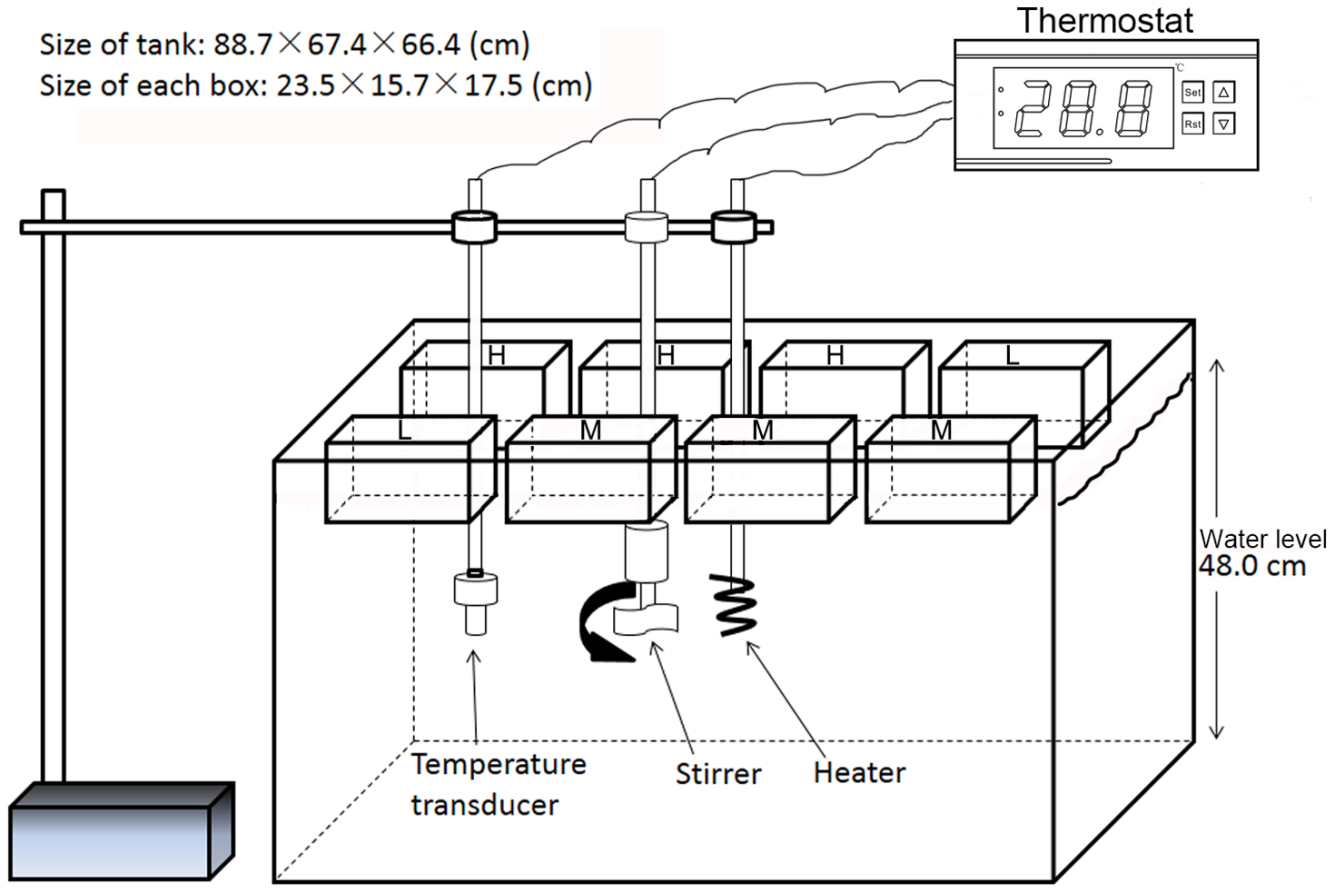
Diagrams illustrating the general arrangement of incubation and heating elements. Water containing a natural phytoplankton community from Taihu was incubated in boxes floating on the surface of large water-filled tanks equipped with a heating system. The heater was put in the middle of the tank with a stirrer beside it. A temperature transducer was placed a bit farther from the heater. The heating process was controlled by a thermostat to maintain a constant water temperature in the tank. There were six such tanks used in our experiment. There was no heating system in the first tank. The others were heated to 12°C, 14°C, 16°C, 18°C and 20°C. There were three nutrients levels, labeled as L, M and H, in each tank.

Laboratory experiments were conducted in the laboratory from January 13–25, 2013. There were three nutrient levels (low: TN∼2 mg·L^−1^, TP∼0.06 mg·L^−1^; medium: TN∼7 mg·L^−1^, TP∼0.3 mg·L^−1^ and high: TN∼10 mg·L^−1^, TP∼1 mg·L^−1^; L, M and H were used for short) and six water temperature levels (unheated, 12°C, 14°C, 16°C, 18°C and 20°C; A, B, C, D, E and F for short, respectively) in our experiment. Low nutrient concentrations treatments were used as nutrient control treatments in which no extra nutrients were added. We selected these temperatures because the mean water temperature in spring was 16°C in Lake Taihu according to our monitoring data. Six large tanks (∼400 L) were used as water-bath heaters (Figure 2). Five of them (labeled as B, C, D, E, and F) were heated to different temperatures using immersion heaters, and the temperatures were controlled by thermostats (CHD702, China). The temperature-control-system operated with a precision of ±0.2°C and functioned without any interruption or breakdown. The sixth tank (labeled A) was not heated as a temperature-treatment control, and the water temperature was recorded using a YSI 6600V2 probe (Yellow Springs Instruments, USA).

Incubations were conducted in 4 L plastic boxes; 8 boxes were floated in each tank (Figure 2). In the “L” treatment, no nutrients were added. In the “M” and “H” treatments, KNO_3_ and K_2_HPO_4_ were added to specific concentrations. Both the “M” and “H” treatments consisted of three replicates, and the “L” treatments consisted of two replicates because of the limited space in each tank. Six fluorescent lamps (Power rating: 11 W) were attached to the roof to prolong the illumination time.

All 48 boxes were filled with 4 L of lake water from Meiliang Bay on the morning of January 13, 2013. Samples were taken from each box at 13:00 on a daily basis. Concentrations of cyanobacteria, Chlorophyta and Bacillariophyta and Chl *a* were estimated using Phyto-PAM (Walz, Germany). Phyto-PAM is a non-intrusive method [34] that measures fluorescence at four wavelength signals (470 nm, 520 nm, 645 nm and 665 nm) and therefore shows the contribution of various types of pigments [35]. The Chl *a* data for cyanobacteria, green algae and diatoms were calculated from the original 4-channel fluorescence data by an on-line deconvolution routine, based on previously stored “reference excitation spectra”. Such “spectra”, which consist of only four points at 470, 535, 620 and 650 nm, can be readily measured under “Reference” for any pure algae culture. However, in any case, the differences between cyanobacteria, green algae and diatoms are sufficiently large to allow at least a coarse differentiation, even if the particular species contained in a sample were not identified [36]. The references used for cyanobacteria in our experiments were obtained according to pure *Microcystis aeruginosa* culture, references for green algae were obtained from *Scenedesmus obliquus* and diatom references were obtained from *Aulacoseira granulata*. All the species isolates were obtained from the Freshwater Algae Culture Collection of Institute of Hydrobiology, Chinese Academy of Sciences, which located in Wuhan, Hubei Province. Phytoplankton community structure was determined based on microscopic observations, using the same procedures as described for the field studies, at the beginning and end of the experiment.

### Data analysis

The temporal trend in conductivity was evaluated for significance using the non-parametric Mann-Kendall test [37]. A correlation analysis (Pearson correlation, *r*) was performed between conductivity and the other environmental variables.

Ordination was used to quantify the influence of environmental variables on phytoplankton community succession in spring. Two data matrices were used. One included the phytoplankton biovolume, and a second one included the environmental variables. To run the analysis, species that occurred in more than 40 of the 80 total samples were included. Before conducting RDA, we analyzed the historical dynamics of spring phytoplankton community between 1992 and 2012 by using two indexes, the Bray & Curtis (BC, dissimilarity coefficient) index and Sørensen coefficient (non metric coefficient). Results (Table S1 and Table S2) indicated that the dynamics of spring phytoplankton community were mainly influenced by biomass variation; no new species arise and no initial resident species dying out. In this case, only include those species that occurred in more than 40 of the 80 total samples would not introduce too much bias due to omit rare species. The environmental matrix included water temperature in May (WT), cumulative water temperature in spring (CWT), mean conductivity in spring (Cond) and mean trophic state index (TSI). Here we used WT and CWT as proxies of climate change, and Cond and TSI as proxies of trophic state. Species data were square root transformed, and environmental variables were center and standardization transformed before analyzing. Detrended correspondence analysis was used to determine the maximum gradient length of species metrics, which was 2.9, indicating that linear methods would be appropriate [38]. All canonical axes were used to evaluate the significant variables under analysis by means of a Monte Carlo test (1000 permutations). There were no colinearities among the environmental variables (variance inflation factors <20). The variance partitioning technique (partial ordination analysis) was applied to separate the effects of climatic variables and trophic state and their interactive effects on phytoplankton community [39].

Treatments effects, and interactions between them, on phytoplankton community shifts in our experiment were determined by univariate analysis of variance (ANOVA) using general linear models (the procedures followed [40]). The ratio calculated follow Equation 2 was used as the dependent variable. The proportion of cyanobacteria was small, and hence, it was omitted in our calculation. Incubation days, water temperatures and total nitrogen concentrations were used as independent variables. The interactive effect of water temperature and total nitrogen was included in our analysis as well.
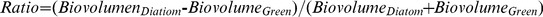
(2)Where *Ratio*>0 means phytoplankton community was dominated by diatom, *Ratio*<0 means it was dominated by green algae, and when the ratio around 0 means they were diatom and green algae co-dominated.

ANOVA provides an extremely powerful and useful tool for statistical tests of factors and their interactions in experiments [41]. However, it was not appropriate for comparing the contribution of each treatment. Hence, partial regression coefficients [42] of linear regression models both with and without interactive factor [43] were used to compare the contributions of treatments and their interactive effects to the ratio. The interactive effect was calculated by TN× water temperature.

The Mann-Kendall test was performed with R statistical software [44] using the *Kendall* package for R. The correlation analyses, univariate analysis of variance and linear regression were conducted using IBM SPSS Statistics 20. Ordination analyses were performed by using Canoco 5. Graphs were draw with OriginPro 8.0.

## Results

### Long term changes in environmental variables

**Figure 3:**
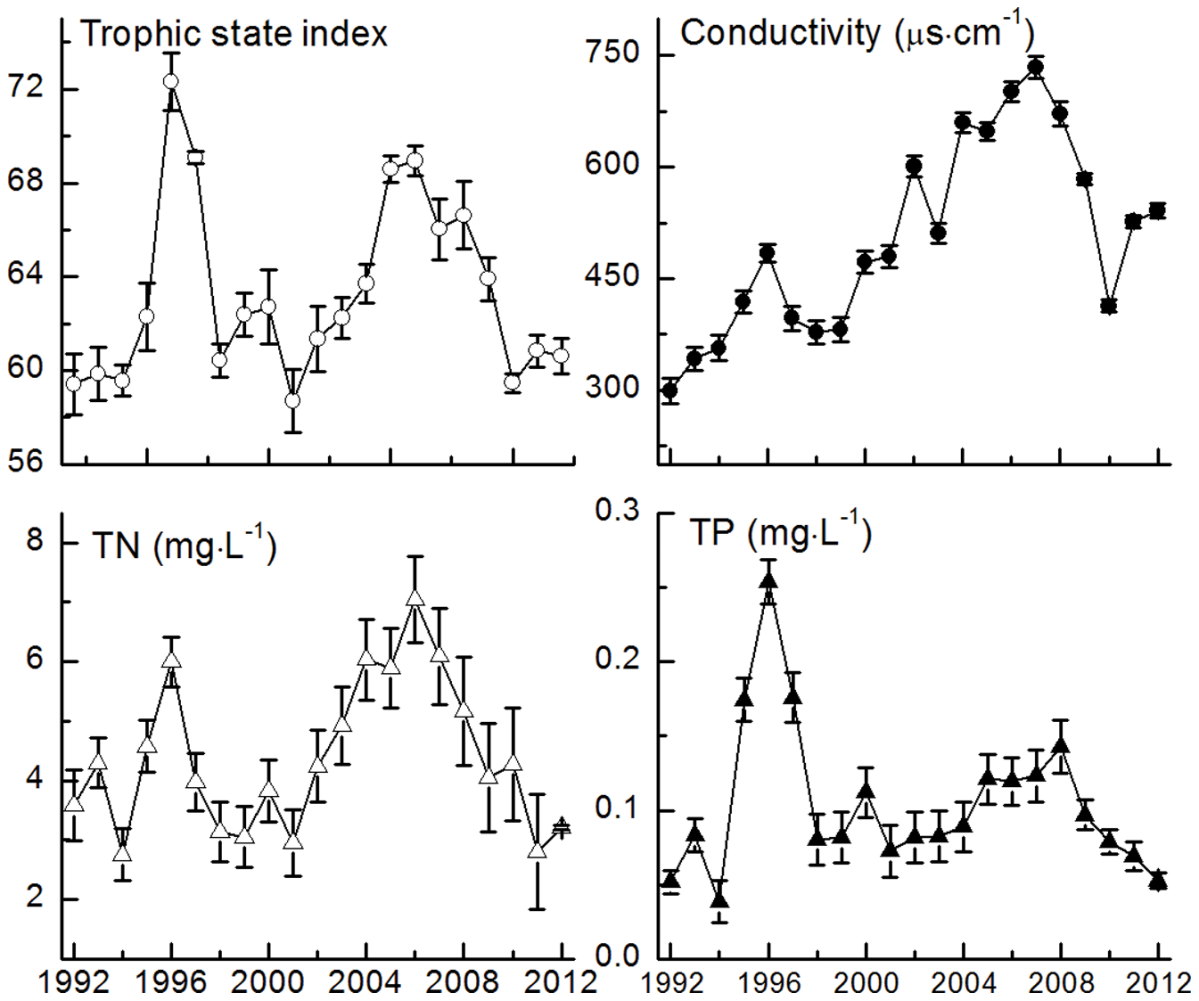
Trends of the physical and chemical variables. All data are shown as the mean ± SD.

Conductivity exhibited a significant increasing trend before 2007 (τ = 0.9, *p*<0.001). It has decreased since 2008 (Figure 3). The variation in conductivity was strongly and significantly related to the variations in Cl^−^, SO_4_^2−^ and K^+^ (Pearson correlation *r*>0.5, *p*<0.01). It was also related to those of NO_2_^−^, NO_3_^−^, Na^+^, Ca^2+^ and NH_4_^+^ (*p*<0.01) (Table 1).

**Table 1:** Pearson correlations between conductivity and main ions.

	Cl^−^	SO_4_^2−^	K^+^	NO_2_^−^	NO_3_^−^	Na^+^
Pearson Correlation	0.8**	0.77**	0.5**	0.46**	0.43**	0.4**
Samples number	176	165	156	182	182	156

	Ca^2+^	NH_4_^+^	Si	Mg^2+^	F^−^	PO_4_^3−^
Pearson Correlation	0.36**	0.33**	−0.25**	0.09	−0.12	0.01
Samples number	165	182	156	165	96	179

** Correlation is significant at the 0.01 level (2-tailed).

Over the past 20 years, nutrient concentrations during spring in Meiliang Bay were 0.1±0.05 mg·L^−1^ for TP and 4.3±1.2 mg·L^−1^ for TN, respectively. There were two remarkable peaks in N concentrations (Figure 3). The first one occurred in 1996 and another in 2006. There was only one noticeable peak for TP in 1996 (Figure 3). However, the P concentration exhibited an additional small peak in 2008.

### Long term changes in the phytoplankton community

According to the monthly monitoring data, a total of 31 genera of phytoplankton belonging to 6 phyla have been identified during the past two decades (Table S3). Cyanobacteria and Chlorophyta were the two main phyla in late spring in Meiliang Bay during the past twenty years. The next two most abundant phyla were Bacillariophyta (diatom) and Cryptophyta. These four phyla comprised 98% (ranging from 53% to 100% in all the samples) of total phytoplankton biovolume.

**Figure 4:**
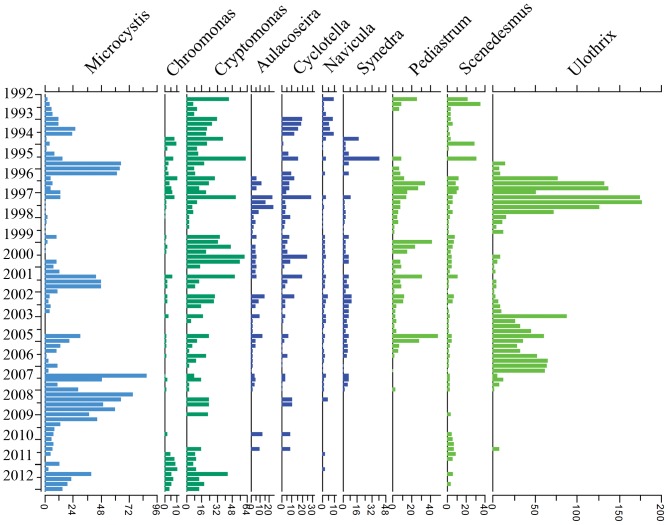
Biovolumes of the most dominant genera in the spring season from 1992–2012. There were no samples in 2004. Biovolume data were square root transformed.

Among the 31 genera identified, only 10 were present in more than half of 80 samples. As showed in Figure 4, *Microcystis* biovolume was high in early 1995, 2001 and 2007–2012. *Cryptomonas* biovolume was high in the early 1990s, 1999–2000 and 2002. *Ulothrix* biovolume was high from 1996–1997 and 2003–2006.

### Ordination

**Figure 5:**
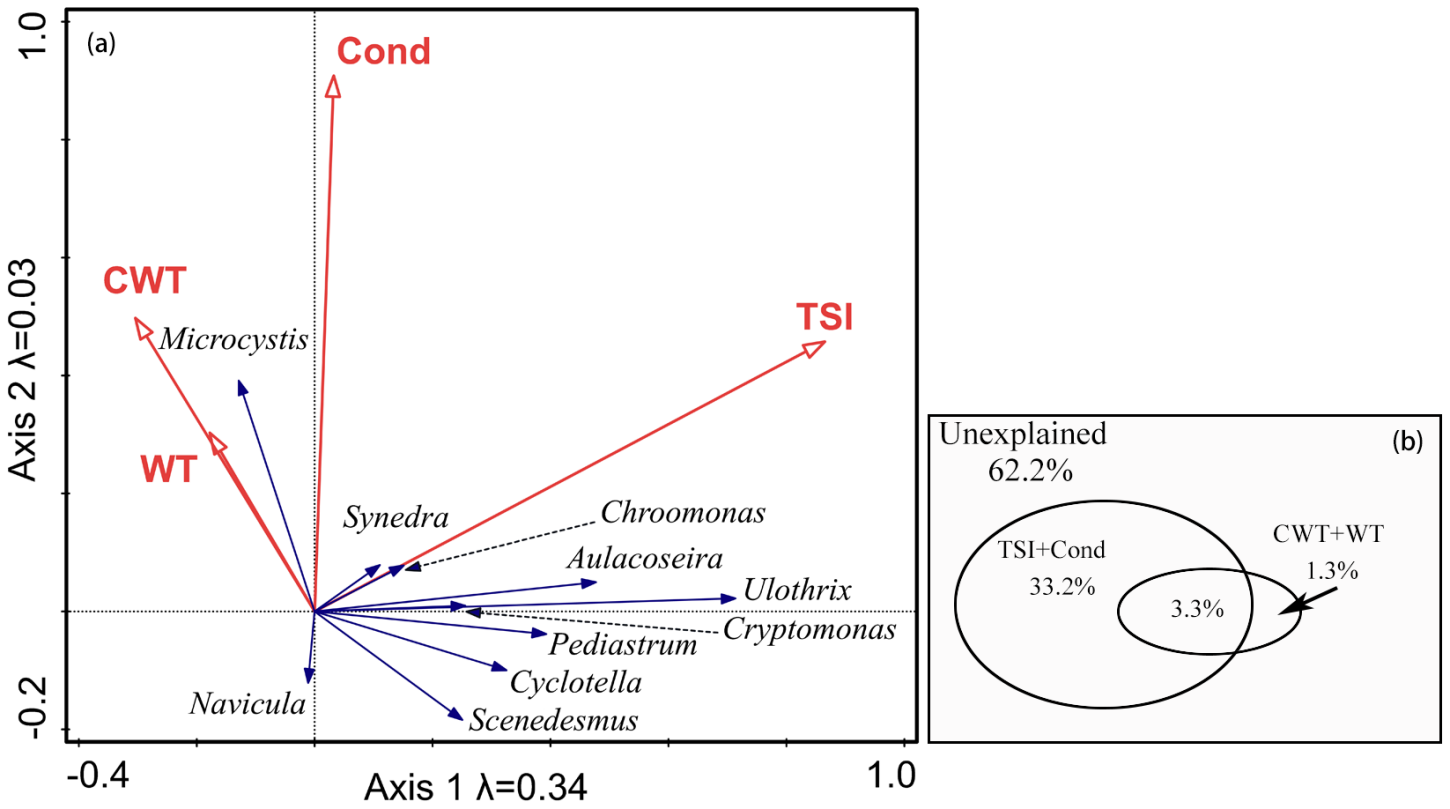
Ordination biplot. (a) Environment variables and dominated species against redundancy analysis axes 1 and 2. (b) Variance partitioning of phytoplankton community, explained by trophic state and climatic variables. See methods for the abbreviations of environmental variables.

The first four RDA axes ordinations accounted for 39.6% of total variance (*p*<0.01), and the first two could explain 36.9%. TSI was the most significant variable that affect phytoplankton community succession in spring (*p*<0.01). TSI alone explained 27.6% of total variance. CWT was the next most significant environmental variable and it explained 3.4% of total variance (*p*<0.05). Cond and WT in May were not significant in our analysis (*p*>0.05). However, they were still significant in our following partial ordination analysis, hence, there were reserved. Accord to Figure 5 (a), most of the dominant genera, for instance, *Ulothrix* and *Aulacoseira*, related to TSI, while *Microcystis* was close related to CWT.

The variance partitioning technique showed that trophic state (tropic state index and conductivity) alone accounted for 33.2% of the total variation (*p*<0.01) (Figure 5(b)). The shared fraction of total variation between trophic state and climatic variables was 3.3% (*p*<0.01). Climatic variable alone accounted for 1.3% of total variation, however, it was statistically not significant (*p*>0.05).

### Experimental results

**Figure 6:**
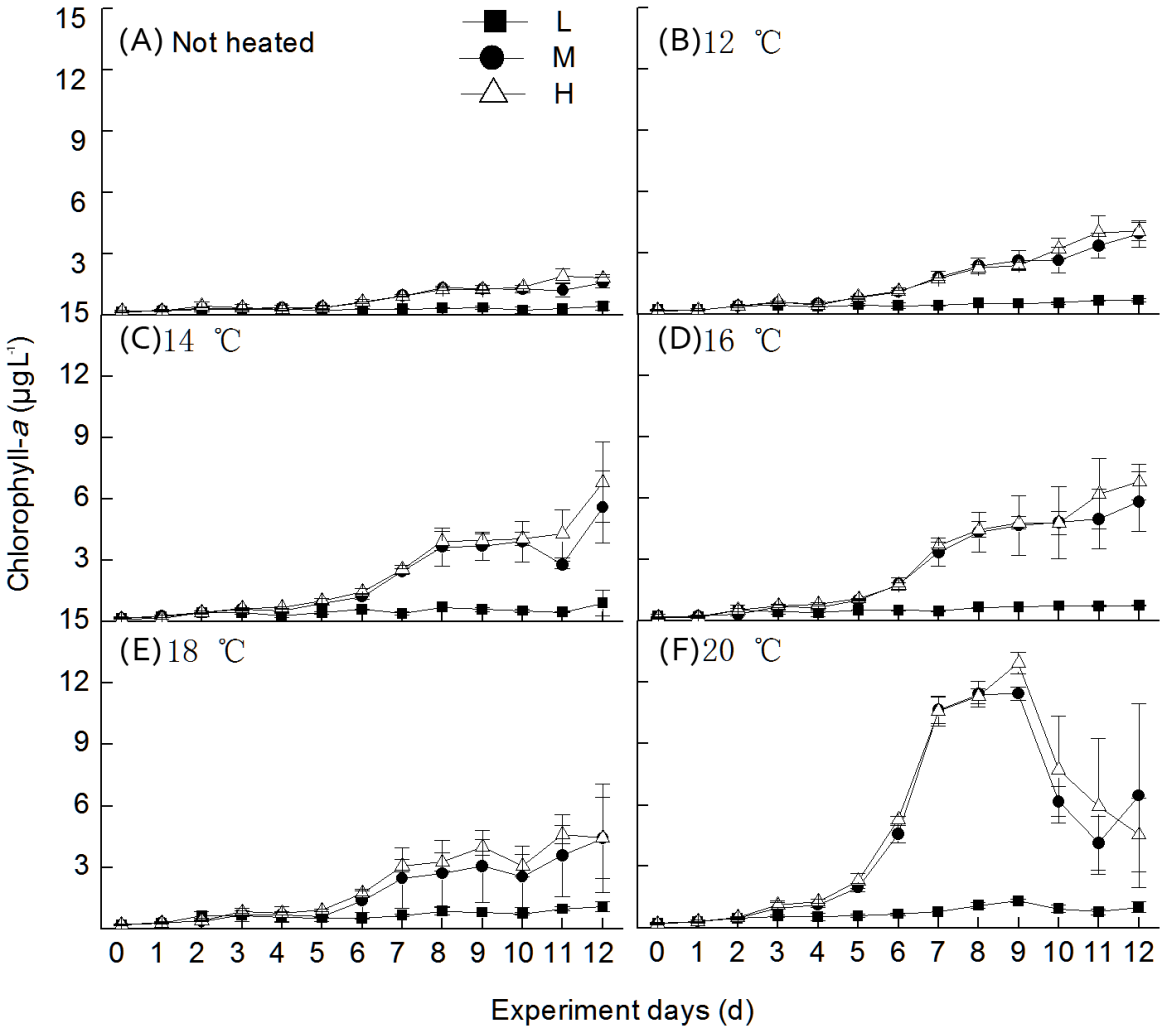
Trends in the total concentration of chlorophyll-*a* in the different water temperature treatments. L, M and H represent the low nutrient concentration treatment (TN∼2 mg·L^−1^, TP∼0.06 mg·L^−1^), medium nutrient concentration treatment (TN∼7 mg·L^−1^, TP∼0.3 mg·L^−1^) and high nutrient concentration treatment (TN∼10 mg·L^−1^, TP∼1 mg·L^−1^), respectively.

The water temperature in Tank A was 9.56±1.5°C during the experimental period. Chl *a* was strongly correlated to both water temperature and nutrient concentrations (Figure 6). Chl *a* concentrations increased slightly beginning on the 2^nd^ day in all treatments. It increased rapidly from the 5^th^ day in treatments B, C, D and E and from the 4^th^ day in F. Total Chl *a* concentrations in the M and H treatments were higher than in the L treatment at each water temperature (*F* = 34.6, *df* = 2, *p*<0.01) (Figure 6). Chl *a* concentrations in the 20°C treatments decreased from the 9^th^ day until the 13^th^ day. Within each nutrient treatment, the Chl *a* concentration increased significantly with water temperature.

**Figure 7:**
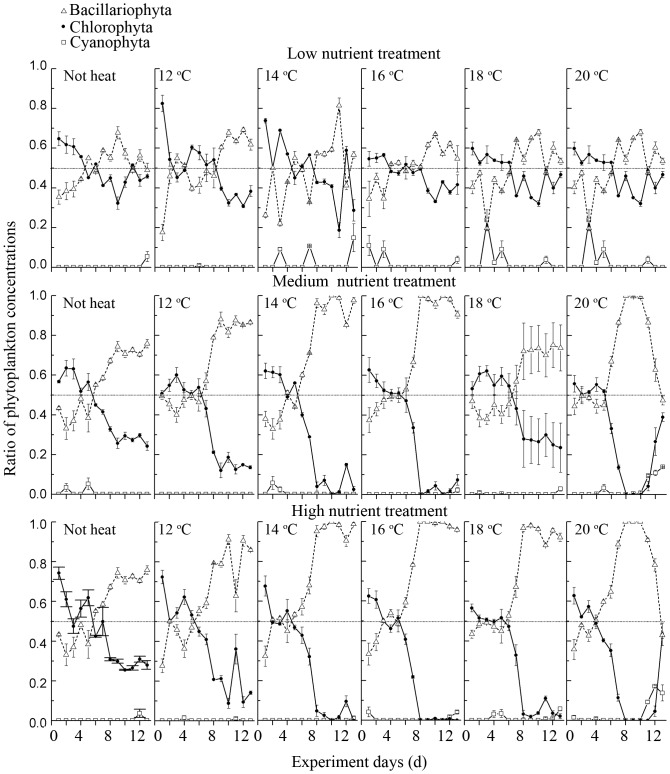
Phytoplankton community succession during the experiment.

Diatoms and green algae were co-dominant at the start of the experiment (Figure 7). The dominant genera were *Planctonema*, *Scenedesmus*, *Cyclotella*, and *Aulacoseira*. In the L treatments, the phytoplankton community consisted of both diatoms and green algae. In the nutrient-added (M and H) treatments, the dominance shifted to diatoms in treatments B, C, D and E on the 7^th^ day and in the F treatments on the 5^th^ day.

Total nitrogen (*F* = 90.1, *df* = 2, *p*<0.01), water temperature (*F* = 9.28, *df* = 5, *p*<0.01) and their interaction (*F* = 4.09, *df* = 10, *p*<0.01) affected phytoplankton community significantly in our experiments according to univariate analysis of variance (*F* = 45.7, *df* = 29, *r* ^2^ = 0.68, *p*<0.01).

A Linear model without interactive effect between TN and water temperature indicated TN affected phytoplankton community variations strongly and significantly (*r* ^2^ was 0.56, *p*<0.01 as showed in Table 2). A linear model with interaction showed that besides incubation days, interactive effect between TN and water temperature was the most significant variable affecting phytoplankton community (*r* ^2^ = 0.57, *p*<0.01 Table 2).

**Table 2:** Results of linear regression models.

	Linear regression	Partial correlations		
	*r* ^2^	TN	WT	TN×WT
Model without interaction	0.56**	0.41**	0.17**	-
Model with interaction	0.57**	−0.04	−0.06	0.14**

Models with and without interaction were both fitted to phytoplankton community (Ratio). The way calculating ratio please refer to methods. TN×WT means the interaction between TN and water temperature.

** *p*<0.01.

-Not included in the model.

## Discussion

In the early 1990s, both the trophic state and conductivity were low in Taihu. Phosphorus was believed to be the primary limiting nutrient in freshwater systems [45]; hence, phosphate-free detergents have been used in the Lake Taihu catchment since 1999 in an effort to reduce P loading. This measure was quite effective [46], and as a result, P concentrations have exhibited only one notable peak in 1996 during the last two decades. However, with no specific focus on nitrogen reduction, N concentrations experienced two notable peaks during the last two decades. The average cumulative water temperature over the last ten years was a bit higher than for the first ten years of our study period. The warming trend in Lake Taihu in spring is reflective of increases in the region's air temperature in spring [18]. Our monitoring data indicated that Cl^−^, SO_4_^2−^, K^+^, NO_2_^−^ and NO_3_^−^ were the main ions that contributed to the variation in conductivity, and the increasing of their concentrations were mainly due to human activities [47]. In the basin, NH_4_Cl, KCl, (NH_4_)_2_SO_4_ and K_2_SO_4_ are most commonly used agricultural fertilizer.

*Microcystis* biomass was positively related to CWT and WT in our study (as indicated in Fig. 5), this was in accordance with previous studies and conclusions [6], [14], [15], [48]. However, when TP and TN concentrations reached their first peaks in 1996 and 1997, *Microcystis* became less dominant and green algae appeared at a very high percentage (as shown in Figs. 3 and Figure 4 in 1996–2000). Chlorophytes dominated again from 2003 to 2006 when nutrient concentrations were high (Figs. 3 and 4). In our experiments, the dominant phytoplankton taxa remained consistent at all water temperatures in the treatments with no nutrient enrichment (Figure 7). Dominant taxa shifted to diatoms in all nutrient-add treatments. Although, there is currently no consensus within the limnological community about the relative importance of nutrients and temperature in driving phytoplankton community dynamics, long-term monitoring data indicated that nutrients exert a stronger control than water temperature over the composition of phytoplankton communities in spring in Lake Taihu (Figure 5) and this was confirmed by our laboratory experiments (Table 2). However, there was still a gap between field monitoring data and laboratory experiments in our study. Green algae dominated in Meiliang Bay when nutrient concentrations were high, while it was diatom dominated in the nutrient-added treatments in our lab experiment. This may reflect the fact that diatoms possess heavy siliceous walls which render them particularly susceptible to sinking losses in the field [49]. However, the losses did not happened in our experiments. Besides, diatoms are generally fast-growing species under non-limiting conditions [50] and thus would respond quickly in a short time during our incubation. In addition, our experiments were carried out during winter and illumination was not as strong as in spring in Lake Taihu. However, diatoms have a higher inherent growth rate compared to other algae because they have a higher photosynthetic capacity due to a higher chlorophyll content inside [51]. Other factors, such as grazing [52], [53] and mixing condition [54], [55], which were not significant in our experiments, may potentially affect phytoplankton community structure responses. These interpretive problems have likewise affected previous studies, e.g., [56], [57], and this should serve as a reminder that extrapolating the laboratory results to the natural environment should be treated with caution. In our study, laboratory experiments failed to exactly predict the dominant species in Lake Taihu. However, the results clearly indicated that in general, phytoplankton community succession is more sensitive to nutrient concentrations than temperature shifts.

Compared to the published literature on Lake Taihu, which mainly focused on single species or Chl *a* as a proxy for phytoplankton (e.g. [14]–[17] and so on), our results demonstrated that nutrient concentrations became the principal factor that affect phytoplankton at the community level. Badeck et al. [58] found that the correction between satellite and ground phenology estimates was higher when taxa composition is known or homogenous, suggesting a compositional role. It is likely that, in many cases, mismatches might result from not considering the importance of shifts in community composition, especially with respect to dominant taxa [19].

Our study was carried out in subtropical lake and these results are not the exception when compared to lakes globally. Phytoplankton community changes among European peri-alpine lakes over 25 years were mainly driven by variation in phosphorus concentrations, and it was also affected by warmer winters [59]. Phytoplankton composition in 35 lakes ranging from the subtropics to the temperate zone in North America and Europe is primarily driven by nutrient loading, while climate change effects are less detectable [60]. A study based on >1000 US lakes found that the most important explanatory driver for phytoplankton (Chl *a* was used as proxy) was nutrient availability [11]. In addition, a paleolimnological study also found that nutrients played a more crucial role than water temperature in controlling the diatom community over the past 60 years in Esthwaite Water, UK [61]. Results of one phytoplankton community model showed that changes in nutrient loading generally had a greater effect on the composition of phytoplankton communities than changes in water temperature [62]. Based on these cases, it appears that when compared to water temperature, nutrients availability is the main driver for phytoplankton community structure variation.

There is growing concern that interactions between climate warming and eutrophication affect aquatic ecosystems globally. A previous study reported that changes on photosynthesis, respiration and growth of natural phytoplankton communities were strongly related to interactions of temperature and nutrient availability [63]. Rhee and Gothan [64] argued that as temperature increases, so does the demand for nutrients in phytoplankton growth as well. Our results also showed that interactions between nutrients and water temperature have a strong effect on phytoplankton community succession in Lake Taihu (Fig. 5). The spring phytoplankton community variation in Lake Taihu was mediated by changes in nutrient concentrations, and this effect was strongly enhanced by high water temperature. This was also reported in other studies, e.g., [59]. Studies of climate–nutrient interactions in lakes, streams and wetlands in the Euro-limpacs project and elsewhere have shown that warming is likely to exacerbate symptoms of eutrophication in freshwaters [65]. A study by Rigosi et al. [11] includes an in depth discussion on the interaction of climate warming and eutrophication on phytoplankton, although it was mainly focused on cyanobacterial dominance. They found that the interaction between warming and eutrophication is dependent on trophic state, especially how it promote cyanobacterial blooms. This discussion also partly support our earlier conclusion. Although with only a single case we cannot address such analogous conclusions, our results appear to support their conclusion. Lake Taihu is eutrophic and the interaction between water temperature and nutrient availability was significant. Most of the studies that related to interaction of climate variables and nutrients availability were carried out in eutrophic lakes, and still we cannot definitively determine the mechanisms driving this result, we hypothesize that this may be because species response differently to environmental variable variations, and different species will dominate among lakes having differing trophic states.

In conclusion, our results show that nutrients concentrations are the dominant environmental factors that influence phytoplankton community successional patterns during bloom development in Lake Taihu. However, interactions between nutrients concentrations and water temperature plays an additional, and thus for unexplored, variable involved in structuring phytoplankton communities, including taxa involved in bloom formation.

## Supporting Information

Table S1 **Similarity of species composition test by B&C indexes among samples.**
(DOCX)

Table S2 **Similarity of species composition test by Sørensen coefficients (non metric coefficient) among samples.**
(DOCX)

Table S3 **Name list of the genera found in spring in Lake Taihu.**
(DOCX)

Table S4 **Biomass of cyanobacteria, green algae, diatom, and chlorophyll** ***a*** **concentration in laboratory experiments.**
(DOCX)
