# Supplementary material for: Effects of Nutrients, Temperature and Their Interactions on Spring Phytoplankton Community Succession in Lake Taihu, China
Source: PLoS One. 2014 Dec 2;9(12):e113960. doi: 10.1371/journal.pone.0113960 (PMC4252073; doi:10.1371/journal.pone.0113960)
Supplement: Table S2 — Similarity of species composition test by Sørensen coefficients (non metric coefficient) among samples. (DOCX) [file pone.0113960.s002.docx]

**Table S2:** Similarity of species composition test by Sørensen coefficients (non metric coefficient) among samples.

| 1993 | 1993 |  |  |  |  |  |  |  |  |  |  |  |  |  |  |  |  |  |
| --- | --- | --- | --- | --- | --- | --- | --- | --- | --- | --- | --- | --- | --- | --- | --- | --- | --- | --- |
| 1994 | 0.8 | 1994 |  |  |  |  |  |  |  |  |  |  |  |  |  |  |  |  |
| 1995 | 0.6 | 0.8 | 1995 |  |  |  |  |  |  |  |  |  |  |  |  |  |  |  |
| 1996 | 0.5 | 0.5 | 0.6 | 1996 |  |  |  |  |  |  |  |  |  |  |  |  |  |  |
| 1997 | 0.5 | 0.6 | 0.7 | 0.7 | 1997 |  |  |  |  |  |  |  |  |  |  |  |  |  |
| 1998 | 0.5 | 0.6 | 0.7 | 0.7 | 0.8 | 1998 |  |  |  |  |  |  |  |  |  |  |  |  |
| 1999 | 0.5 | 0.7 | 0.7 | 0.6 | 0.8 | 0.8 | 1999 |  |  |  |  |  |  |  |  |  |  |  |
| 2000 | 0.5 | 0.5 | 0.7 | 0.6 | 0.7 | 0.8 | 0.7 | 2000 |  |  |  |  |  |  |  |  |  |  |
| 2001 | 0.6 | 0.7 | 0.8 | 0.6 | 0.8 | 0.8 | 0.7 | 0.7 | 2001 |  |  |  |  |  |  |  |  |  |
| 2002 | 0.6 | 0.6 | 0.8 | 0.7 | 0.8 | 0.8 | 0.7 | 0.8 | 0.9 | 2002 |  |  |  |  |  |  |  |  |
| 2003 | 0.5 | 0.6 | 0.8 | 0.7 | 0.8 | 0.8 | 0.8 | 0.7 | 0.8 | 0.7 | 2003 |  |  |  |  |  |  |  |
| 2005 | 0.5 | 0.6 | 0.8 | 0.6 | 0.7 | 0.8 | 0.8 | 0.8 | 0.8 | 0.9 | 0.7 | 2005 |  |  |  |  |  |  |
| 2006 | 0.4 | 0.6 | 0.8 | 0.6 | 0.7 | 0.8 | 0.8 | 0.8 | 0.8 | 0.8 | 0.8 | 0.9 | 2006 |  |  |  |  |  |
| 2007 | 0.5 | 0.5 | 0.7 | 0.6 | 0.8 | 0.9 | 0.7 | 0.8 | 0.8 | 0.9 | 0.7 | 0.9 | 0.9 | 2007 |  |  |  |  |
| 2008 | 0.7 | 0.8 | 0.5 | 0.4 | 0.4 | 0.3 | 0.5 | 0.4 | 0.5 | 0.5 | 0.4 | 0.5 | 0.5 | 0.4 | 2008 |  |  |  |
| 2009 | 0.7 | 0.6 | 0.5 | 0.4 | 0.3 | 0.3 | 0.3 | 0.3 | 0.4 | 0.4 | 0.3 | 0.3 | 0.3 | 0.2 | 0.5 | 2009 |  |  |
| 2010 | 0.6 | 0.8 | 0.6 | 0.6 | 0.5 | 0.5 | 0.6 | 0.4 | 0.6 | 0.5 | 0.5 | 0.6 | 0.6 | 0.5 | 0.6 | 0.6 | 2010 |  |
| 2011 | 0.6 | 0.6 | 0.5 | 0.5 | 0.5 | 0.4 | 0.5 | 0.4 | 0.5 | 0.4 | 0.5 | 0.5 | 0.5 | 0.5 | 0.4 | 0.5 | 0.6 | 2011 |
| 2012 | 0.5 | 0.6 | 0.5 | 0.4 | 0.3 | 0.3 | 0.3 | 0.3 | 0.5 | 0.5 | 0.3 | 0.5 | 0.5 | 0.4 | 0.5 | 0.6 | 0.7 | 0.7 |

Yellow indicated that Sørensen coefficient ≥ 0.7 (the two sample share highly accordant species composition), sandybrown indicated that 0.7 > Sørensen coefficient ≥ 0.6 (the two sample share accordant species composition), orange indicated that 0.6 > Sørensen coefficient ≥ 0.5 (the two sample share similar species composition), and green indicated that Sørensen coefficient ≤ 0.5 (species composition was different between the two samples).
