# Supplementary material for: Effects of Nutrients, Temperature and Their Interactions on Spring Phytoplankton Community Succession in Lake Taihu, China
Source: PLoS One. 2014 Dec 2;9(12):e113960. doi: 10.1371/journal.pone.0113960 (PMC4252073; doi:10.1371/journal.pone.0113960)
Supplement: Table S3 — Name list of the genera found in spring in Lake Taihu. (DOCX) [file pone.0113960.s003.docx]

**Table S3:** Name list of the genera found in spring in Lake Taihu

| **Year** | **1992** | **1993** | **1994** | **1995** | **1996** | **1997** | **1998** | **1999** | **2000** | **2001** | **2002** | **2003** | **2005** | **2006** | **2007** | **2008** | **2009** | **2010** | **2011** | **2012** |
| --- | --- | --- | --- | --- | --- | --- | --- | --- | --- | --- | --- | --- | --- | --- | --- | --- | --- | --- | --- | --- |
| *Anabeana* spp. | **+** | **++** | **+** | **++** |  |  |  |  |  | **+** | **+** |  | **+** |  |  | **++** | **++** | **++** |  | **+** |
| *Aphanizomenon* sp. |  |  |  |  |  |  | **+** |  | **+** |  |  |  |  |  |  |  |  |  |  |  |
| *Chroococcus* sp. |  |  |  |  | **+** |  |  |  |  |  |  |  |  |  |  |  |  |  |  |  |
| *Dactylococcopsis* sp. |  |  |  |  |  |  |  |  |  |  |  |  |  |  | **+** |  |  |  |  |  |
| *Microcystis* spp. | **++** | **++** | **+** | **++** | **+** |  |  | **+** | **++** | **++** | **+** |  | **++** | **+** | **++** | **++** | **++** | **+** | **+** | **++** |
| *M*. *aeruginosa* |  |  |  |  |  | **+** | **+** |  |  |  |  |  |  |  |  |  |  |  |  |  |
| M. *flos-aquae* |  |  |  |  |  | **+** | **+** |  |  |  |  |  |  |  |  |  |  |  |  |  |
| M. *wesenbergii* |  |  |  |  |  | **+** |  |  |  |  |  |  |  |  |  |  |  |  |  |  |
| M. *incerta* |  |  |  |  |  |  | **+** |  |  |  |  |  |  |  |  |  |  |  |  |  |
| *Merismopedia* sp. |  |  |  |  |  | **+** |  | **+** |  |  |  |  |  |  |  |  |  |  | **+** |  |
| *Planktothrix* spp. | **+** | **++** |  |  |  | **+** | **+** |  |  | **+** | **+** |  |  |  | **+** |  |  |  |  |  |
| *Chroomonas acuta* |  |  | **+** | **+** | **+** | **+** | **+** | **+** |  | **+** | **+** | **+** | **+** | **+** | **+** |  |  | **+** | **++** | **+** |
| *Cryptomons ovata* | **++** | **++** | **++** | **++** | **++** | **++** | **+** | **++** | **++** | **++** | **++** | **+** | **++** | **++** | **+** | **++** | **++** | **+** | **++** | **++** |
| *C*. *erosa* |  |  |  | **++** | **++** | **++** | **+** | **++** | **++** | **++** | **++** | **+** | **+** | **+** | **+** |  |  |  |  |  |
| *Ceratium hirundinella* |  |  |  |  |  |  |  |  |  |  |  |  |  |  |  |  |  |  |  |  |
| *Euglena* spp. | **++** |  | **+** |  |  |  |  |  |  |  |  |  |  |  |  |  |  |  |  |  |
| *E*. *acus* |  |  |  |  |  |  |  | **+** |  |  |  |  | **+** |  |  |  |  |  |  |  |
| *E*. *oxyuris* |  |  |  |  |  |  |  |  |  |  |  |  | **+** | **+** |  | **++** |  |  |  |  |
| *Asterionella* sp. |  |  |  |  |  |  |  |  |  |  |  |  |  |  |  |  |  |  |  | **+** |
| *Aulacoseira* *granulata* | **+** |  |  |  | **+** | **++** | **+** | **+** | **+** | **+** | **+** | **+** | **+** | **+** | **+** |  |  | **+** |  |  |
| *A*. *g*.var. *angrstissima* |  |  |  |  |  | **++** | **+** | **+** | **+** |  | **+** | **+** | **+** | **+** | **+** |  |  |  |  |  |
| *A*. *islandica* |  |  |  |  |  | **+** | **+** |  |  |  |  |  |  |  |  |  |  |  |  |  |
| *Cyclotella* spp. |  | **++** | **+** | **++** | **+** | **++** | **+** | **+** | **++** | **++** | **+** | **+** | **+** | **+** | **+** | **+** |  | **+** |  |  |
| *Fragilaria* spp. |  |  | **++** | **++** |  | **+** | **+** | **+** | **+** | **+** | **+** | **+** | **+** | **+** | **+** |  |  |  |  |  |
| *Navicula* spp. | **+** | **+** | **+** | **+** |  | **+** | **+** | **+** | **+** | **+** | **+** | **+** | **+** | **+** | **+** | **+** |  |  | **+** |  |
| *Surirella robusta* |  |  |  |  |  | **+** |  |  | **+** |  |  |  |  |  |  |  |  |  |  |  |
| *Actinastrum* sp. |  |  |  |  |  |  |  |  |  |  |  |  |  |  |  |  | **+** |  |  |  |
| *Ankistrodesmus falcatus* |  |  |  |  |  |  |  | **+** |  |  |  |  |  |  |  |  |  |  |  |  |
| *Closterium* spp. |  |  |  |  | **+** | **+** | **+** |  |  |  | **+** |  | **+** |  | **+** |  |  |  |  |  |
| *Closteriopsis* sp. |  |  |  | **+** |  |  |  |  | **+** |  | **+** |  | **+** | **+** | **+** |  |  |  |  |  |
| *Coelastrum* sp. |  |  |  |  |  |  | **+** | **+** | **+** |  |  |  | **+** |  | **+** |  |  |  |  |  |
| *Crucigenia* spp. |  |  |  | **+** | **+** | **+** | **+** | **+** | **+** | **+** | **+** | **+** | **+** | **+** | **+** |  |  |  |  |  |
| *Cocconeis* sp. |  |  |  |  |  |  |  |  |  |  |  |  | **+** | **+** | **+** |  |  | **+** | **+** | **+** |
| *Pediastrum* spp. | **++** |  |  | **+** | **++** |  |  |  |  |  |  |  |  |  |  |  |  |  |  |  |
| *P*. *duplex* |  |  |  |  |  | **+** | **+** | **++** | **+** | **++** | **+** | **+** | **++** | **+** |  |  |  |  |  |  |
| *P*. *simplex* |  |  |  |  |  | **+** | **+** |  |  | **+** | **+** | **+** | **++** | **+** | **+** |  |  |  |  |  |
| *P*. *s*.var.*duodenarium* |  |  |  |  |  |  | **+** |  |  |  |  |  |  |  |  |  |  |  |  |  |
| *P*. *tetras* |  |  |  |  |  |  | **+** |  |  |  |  |  |  |  |  |  |  |  |  |  |
| *Scenedesmus* spp. | **++** | **+** | **++** |  |  |  |  |  |  |  |  |  |  |  |  |  | **+** | **+** | **+** |  |
| *S*. *obliquus* |  |  |  | **++** | **+** | **+** | **+** | **+** | **+** | **+** | **+** | **+** | **+** | **+** | **+** |  |  |  |  |  |
| *S*. *bijuga* |  |  |  |  |  |  | **+** | **+** | **+** | **+** | **+** | **+** | **+** | **+** | **+** |  |  |  |  |  |
| *S*. *dimorphus* |  |  |  | **+** | **+** | **+** | **+** | **+** | **+** | **+** | **+** | **+** | **+** | **+** | **+** |  |  |  |  |  |
| *S*. *arcuatus* |  |  |  |  |  |  | **+** | **+** | **+** | **+** | **+** | **+** | **+** | **+** | **+** |  |  |  |  |  |
| *S*. *quadricauda* |  |  |  | **++** | **+** | **+** | **+** | **+** | **+** | **+** | **+** | **+** | **+** | **+** | **+** |  |  |  |  | **+** |
| *Schroederia* sp. |  |  |  |  |  |  |  |  |  | **+** | **+** |  | **+** | **+** | **+** | **+** |  |  |  | **+** |
| *Staurastrum* spp. | **+** |  |  |  |  |  |  |  |  |  |  |  |  |  |  |  |  |  |  |  |
| *Tetraedron* spp. |  |  |  |  |  |  | **+** |  | **+** | **+** | **+** |  | **+** | **+** | **+** |  |  |  |  |  |
| *Ulothrix* spp. |  |  |  | **++** | **++** | **++** | **++** |  | **+** | **+** | **+** | **++** | **++** | **++** | **+** |  |  | **+** |  |  |

“+”: occurred. “++”: A lot of.
