# Supplementary material for: Effects of Nutrients, Temperature and Their Interactions on Spring Phytoplankton Community Succession in Lake Taihu, China
Source: PLoS One. 2014 Dec 2;9(12):e113960. doi: 10.1371/journal.pone.0113960 (PMC4252073; doi:10.1371/journal.pone.0113960)
Supplement: Table S4 — Biomass of cyanobacteria, green algae, diatom, and chlorophyll a concentration in laboratory experiments. (DOCX) [file pone.0113960.s004.docx]

**Table S4:** Biomass of cyanobacteria, green algae diatom, and chlorophyll *a* concentration in laboratory experiments

| **Date** | **Sample** | **c(cyanobacteria)** | **c(green algae)** | **c(diatom)** | **Chl a (ug/L)** |
| --- | --- | --- | --- | --- | --- |
| 2013/1/13 | AH-1 | 0 | 0.15 | 0.06 | 0.21 |
| 2013/1/13 | AH-2 | 0 | 0.12 | 0.06 | 0.18 |
| 2013/1/13 | AH-3 | 0 | 0.22 | 0.04 | 0.26 |
| 2013/1/13 | AM-1 | 0 | 0.1 | 0.07 | 0.17 |
| 2013/1/13 | AM-2 | 0 | 0.09 | 0.07 | 0.16 |
| 2013/1/13 | AM-3 | 0 | 0.11 | 0.09 | 0.20 |
| 2013/1/13 | AL-1 | 0 | 0.08 | 0.06 | 0.14 |
| 2013/1/13 | AL-2 | 0 | 0.13 | 0.05 | 0.18 |
| 2013/1/13 | BH-1 | 0 | 0.2 | 0.04 | 0.24 |
| 2013/1/13 | BH-2 | 0 | 0.12 | 0.07 | 0.19 |
| 2013/1/13 | BH-3 | 0 | 0.12 | 0.05 | 0.17 |
| 2013/1/13 | BM-1 | 0 | 0.1 | 0.08 | 0.18 |
| 2013/1/13 | BM-2 | 0 | 0.09 | 0.09 | 0.18 |
| 2013/1/13 | BM-3 | 0 | 0.07 | 0.08 | 0.15 |
| 2013/1/13 | BL-1 | 0 | 0.21 | 0.02 | 0.23 |
| 2013/1/13 | BL-2 | 0 | 0.14 | 0.05 | 0.19 |
| 2013/1/13 | CH-1 | 0 | 0.13 | 0.04 | 0.17 |
| 2013/1/13 | CH-2 | 0 | 0.13 | 0.04 | 0.17 |
| 2013/1/13 | CH-3 | 0 | 0.06 | 0.06 | 0.12 |
| 2013/1/13 | CM-1 | 0 | 0.18 | 0.06 | 0.24 |
| 2013/1/13 | CM-2 | 0 | 0.09 | 0.08 | 0.17 |
| 2013/1/13 | CM-3 | 0 | 0.07 | 0.05 | 0.12 |
| 2013/1/13 | CL-1 | 0 | 0.16 | 0.05 | 0.21 |
| 2013/1/13 | CL-2 | 0 | 0.1 | 0.04 | 0.14 |
| 2013/1/13 | DH-1 | 0 | 0.12 | 0.06 | 0.18 |
| 2013/1/13 | DH-2 | 0.03 | 0.17 | 0.04 | 0.24 |
| 2013/1/13 | DH-3 | 0 | 0.07 | 0.07 | 0.14 |
| 2013/1/13 | DM-1 | 0 | 0.07 | 0.08 | 0.15 |
| 2013/1/13 | DM-2 | 0 | 0.15 | 0.03 | 0.18 |
| 2013/1/13 | DM-3 | 0 | 0.11 | 0.08 | 0.19 |
| 2013/1/13 | DL-1 | 0.07 | 0.2 | 0.05 | 0.32 |
| 2013/1/13 | DL-2 | 0 | 0.07 | 0.08 | 0.15 |
| 2013/1/13 | EH-1 | 0 | 0.09 | 0.09 | 0.18 |
| 2013/1/13 | EH-2 | 0 | 0.13 | 0.08 | 0.21 |
| 2013/1/13 | EH-3 | 0 | 0.11 | 0.08 | 0.19 |
| 2013/1/13 | EM-1 | 0 | 0.13 | 0.07 | 0.20 |
| 2013/1/13 | EM-2 | 0 | 0.09 | 0.09 | 0.18 |
| 2013/1/13 | EM-3 | 0 | 0.08 | 0.1 | 0.18 |
| 2013/1/13 | EL-1 | 0 | 0.14 | 0.07 | 0.21 |
| 2013/1/13 | EL-2 | 0 | 0.09 | 0.08 | 0.17 |
| 2013/1/13 | FH-1 | 0 | 0.1 | 0.08 | 0.18 |
| 2013/1/13 | FH-2 | 0 | 0.1 | 0.08 | 0.18 |
| 2013/1/13 | FH-3 | 0.01 | 0.17 | 0.04 | 0.22 |
| 2013/1/13 | FM-1 | 0 | 0.08 | 0.09 | 0.17 |
| 2013/1/13 | FM-2 | 0 | 0.1 | 0.1 | 0.20 |
| 2013/1/13 | FM-3 | 0 | 0.14 | 0.06 | 0.20 |
| 2013/1/13 | FL-1 | 0 | 0.09 | 0.08 | 0.17 |
| 2013/1/13 | FL-2 | 0.02 | 0.17 | 0.05 | 0.24 |
| 2013/1/14 | AH-1 | 0 | 0.12 | 0.11 | 0.23 |
| 2013/1/14 | AH-2 | 0 | 0.12 | 0.09 | 0.21 |
| 2013/1/14 | AH-3 | 0 | 0.17 | 0.06 | 0.23 |
| 2013/1/14 | AM-1 | 0 | 0.17 | 0.08 | 0.25 |
| 2013/1/14 | AM-2 | 0 | 0.09 | 0.09 | 0.18 |
| 2013/1/14 | AM-3 | 0.03 | 0.21 | 0.05 | 0.29 |
| 2013/1/14 | AL-1 | 0 | 0.1 | 0.09 | 0.19 |
| 2013/1/14 | AL-2 | 0 | 0.17 | 0.07 | 0.24 |
| 2013/1/14 | BH-1 | 0 | 0.09 | 0.1 | 0.19 |
| 2013/1/14 | BH-2 | 0 | 0.08 | 0.08 | 0.16 |
| 2013/1/14 | BH-3 | 0 | 0.11 | 0.1 | 0.21 |
| 2013/1/14 | BM-1 | 0 | 0.11 | 0.1 | 0.21 |
| 2013/1/14 | BM-2 | 0 | 0.08 | 0.09 | 0.17 |
| 2013/1/14 | BM-3 | 0 | 0.13 | 0.07 | 0.20 |
| 2013/1/14 | BL-1 | 0 | 0.08 | 0.09 | 0.17 |
| 2013/1/14 | BL-2 | 0 | 0.16 | 0.1 | 0.26 |
| 2013/1/14 | CH-1 | 0 | 0.11 | 0.12 | 0.23 |
| 2013/1/14 | CH-2 | 0 | 0.08 | 0.08 | 0.16 |
| 2013/1/14 | CH-3 | 0 | 0.09 | 0.09 | 0.18 |
| 2013/1/14 | CM-1 | 0 | 0.14 | 0.13 | 0.27 |
| 2013/1/14 | CM-2 | 0 | 0.16 | 0.08 | 0.24 |
| 2013/1/14 | CM-3 | 0.06 | 0.23 | 0.06 | 0.35 |
| 2013/1/14 | CL-1 | 0 | 0.12 | 0.12 | 0.24 |
| 2013/1/14 | CL-2 | 0 | 0.12 | 0.12 | 0.24 |
| 2013/1/14 | DH-1 | 0 | 0.15 | 0.05 | 0.20 |
| 2013/1/14 | DH-2 | 0 | 0.08 | 0.08 | 0.16 |
| 2013/1/14 | DH-3 | 0 | 0.12 | 0.09 | 0.21 |
| 2013/1/14 | DM-1 | 0 | 0.1 | 0.11 | 0.21 |
| 2013/1/14 | DM-2 | 0 | 0.14 | 0.11 | 0.25 |
| 2013/1/14 | DM-3 | 0 | 0.19 | 0.09 | 0.28 |
| 2013/1/14 | DL-1 | 0 | 0.15 | 0.1 | 0.25 |
| 2013/1/14 | DL-2 | 0 | 0.08 | 0.08 | 0.16 |
| 2013/1/14 | EH-1 | 0 | 0.12 | 0.12 | 0.24 |
| 2013/1/14 | EH-2 | 0 | 0.17 | 0.13 | 0.30 |
| 2013/1/14 | EH-3 | 0 | 0.13 | 0.14 | 0.27 |
| 2013/1/14 | EM-1 | 0 | 0.13 | 0.12 | 0.25 |
| 2013/1/14 | EM-2 | 0.01 | 0.25 | 0.08 | 0.34 |
| 2013/1/14 | EM-3 | 0 | 0.17 | 0.13 | 0.30 |
| 2013/1/14 | EL-1 | 0 | 0.13 | 0.13 | 0.26 |
| 2013/1/14 | EL-2 | 0 | 0.16 | 0.13 | 0.29 |
| 2013/1/14 | FH-1 | 0 | 0.16 | 0.14 | 0.30 |
| 2013/1/14 | FH-2 | 0 | 0.16 | 0.16 | 0.32 |
| 2013/1/14 | FH-3 | 0 | 0.17 | 0.15 | 0.32 |
| 2013/1/14 | FM-1 | 0 | 0.21 | 0.12 | 0.33 |
| 2013/1/14 | FM-2 | 0 | 0.13 | 0.15 | 0.28 |
| 2013/1/14 | FM-3 | 0 | 0.12 | 0.17 | 0.29 |
| 2013/1/14 | FL-1 | 0.01 | 0.22 | 0.14 | 0.37 |
| 2013/1/14 | FL-2 | 0 | 0.12 | 0.13 | 0.25 |
| 2013/1/15 | AH-1 | 0 | 0.25 | 0.39 | 0.64 |
| 2013/1/15 | AH-2 | 0 | 0.15 | 0.1 | 0.25 |
| 2013/1/15 | AH-3 | 0 | 0.2 | 0.26 | 0.46 |
| 2013/1/15 | AM-1 | 0 | 0.17 | 0.09 | 0.26 |
| 2013/1/15 | AM-2 | 0 | 0.15 | 0.17 | 0.32 |
| 2013/1/15 | AM-3 | 0 | 0.3 | 0.09 | 0.39 |
| 2013/1/15 | AL-1 | 0 | 0.27 | 0.12 | 0.39 |
| 2013/1/15 | AL-2 | 0 | 0.12 | 0.11 | 0.23 |
| 2013/1/15 | BH-1 | 0 | 0.2 | 0.12 | 0.32 |
| 2013/1/15 | BH-2 | 0 | 0.2 | 0.23 | 0.43 |
| 2013/1/15 | BH-3 | 0 | 0.21 | 0.18 | 0.39 |
| 2013/1/15 | BM-1 | 0 | 0.29 | 0.11 | 0.40 |
| 2013/1/15 | BM-2 | 0 | 0.21 | 0.2 | 0.41 |
| 2013/1/15 | BM-3 | 0 | 0.22 | 0.17 | 0.39 |
| 2013/1/15 | BL-1 | 0 | 0.19 | 0.28 | 0.47 |
| 2013/1/15 | BL-2 | 0 | 0.17 | 0.17 | 0.34 |
| 2013/1/15 | CH-1 | 0 | 0.2 | 0.22 | 0.42 |
| 2013/1/15 | CH-2 | 0 | 0.25 | 0.28 | 0.53 |
| 2013/1/15 | CH-3 | 0 | 0.23 | 0.22 | 0.45 |
| 2013/1/15 | CM-1 | 0 | 0.27 | 0.12 | 0.39 |
| 2013/1/15 | CM-2 | 0 | 0.19 | 0.22 | 0.41 |
| 2013/1/15 | CM-3 | 0.04 | 0.37 | 0.16 | 0.57 |
| 2013/1/15 | CL-1 | 0.03 | 0.28 | 0.1 | 0.41 |
| 2013/1/15 | CL-2 | 0.05 | 0.32 | 0.09 | 0.46 |
| 2013/1/15 | DH-1 | 0 | 0.24 | 0.22 | 0.46 |
| 2013/1/15 | DH-2 | 0 | 0.32 | 0.31 | 0.63 |
| 2013/1/15 | DH-3 | 0 | 0.25 | 0.28 | 0.53 |
| 2013/1/15 | DM-1 | 0 | 0.22 | 0.11 | 0.33 |
| 2013/1/15 | DM-2 | 0 | 0.14 | 0.18 | 0.32 |
| 2013/1/15 | DM-3 | 0 | 0.16 | 0.18 | 0.34 |
| 2013/1/15 | DL-1 | 0 | 0.18 | 0.15 | 0.33 |
| 2013/1/15 | DL-2 | 0.13 | 0.42 | 0.17 | 0.72 |
| 2013/1/15 | EH-1 | 0 | 0.18 | 0.18 | 0.36 |
| 2013/1/15 | EH-2 | 0 | 0.23 | 0.2 | 0.43 |
| 2013/1/15 | EH-3 | 0 | 0.19 | 0.2 | 0.39 |
| 2013/1/15 | EM-1 | 0 | 0.15 | 0.08 | 0.23 |
| 2013/1/15 | EM-2 | 0 | 0.16 | 0.14 | 0.30 |
| 2013/1/15 | EM-3 | 0 | 0.36 | 0.17 | 0.53 |
| 2013/1/15 | EL-1 | 0.06 | 0.31 | 0.1 | 0.47 |
| 2013/1/15 | EL-2 | 0.27 | 0.37 | 0.14 | 0.78 |
| 2013/1/15 | FH-1 | 0 | 0.24 | 0.27 | 0.51 |
| 2013/1/15 | FH-2 | 0 | 0.3 | 0.18 | 0.48 |
| 2013/1/15 | FH-3 | 0 | 0.35 | 0.21 | 0.56 |
| 2013/1/15 | FM-1 | 0 | 0.24 | 0.2 | 0.44 |
| 2013/1/15 | FM-2 | 0 | 0.24 | 0.24 | 0.48 |
| 2013/1/15 | FM-3 | 0 | 0.25 | 0.25 | 0.50 |
| 2013/1/15 | FL-1 | 0 | 0.23 | 0.18 | 0.41 |
| 2013/1/15 | FL-2 | 0.02 | 0.28 | 0.19 | 0.49 |
| 2013/1/16 | AL-1 | 0 | 0.17 | 0.13 | 0.30 |
| 2013/1/16 | AL-2 | 0 | 0.18 | 0.15 | 0.33 |
| 2013/1/16 | AM-1 | 0 | 0.16 | 0.17 | 0.33 |
| 2013/1/16 | AM-2 | 0 | 0.2 | 0.15 | 0.35 |
| 2013/1/16 | AM-3 | 0 | 0.17 | 0.17 | 0.34 |
| 2013/1/16 | AH-1 | 0 | 0.39 | 0.16 | 0.55 |
| 2013/1/16 | AH-2 | 0 | 0.14 | 0.16 | 0.30 |
| 2013/1/16 | AH-3 | 0 | 0.17 | 0.16 | 0.33 |
| 2013/1/16 | BL-1 | 0 | 0.22 | 0.22 | 0.44 |
| 2013/1/16 | BL-2 | 0 | 0.2 | 0.22 | 0.42 |
| 2013/1/16 | BM-1 | 0 | 0.41 | 0.22 | 0.63 |
| 2013/1/16 | BM-2 | 0 | 0.24 | 0.3 | 0.54 |
| 2013/1/16 | BM-3 | 0 | 0.26 | 0.28 | 0.54 |
| 2013/1/16 | BH-1 | 0.03 | 0.45 | 0.16 | 0.64 |
| 2013/1/16 | BH-2 | 0 | 0.28 | 0.29 | 0.57 |
| 2013/1/16 | BH-3 | 0 | 0.43 | 0.21 | 0.64 |
| 2013/1/16 | CL-1 | 0 | 0.22 | 0.17 | 0.39 |
| 2013/1/16 | CL-2 | 0 | 0.3 | 0.22 | 0.52 |
| 2013/1/16 | CM-1 | 0 | 0.35 | 0.3 | 0.65 |
| 2013/1/16 | CM-2 | 0 | 0.28 | 0.32 | 0.60 |
| 2013/1/16 | CM-3 | 0 | 0.28 | 0.32 | 0.60 |
| 2013/1/16 | CH-1 | 0 | 0.54 | 0.18 | 0.72 |
| 2013/1/16 | CH-2 | 0 | 0.29 | 0.35 | 0.64 |
| 2013/1/16 | CH-3 | 0 | 0.28 | 0.34 | 0.62 |
| 2013/1/16 | DL-1 | 0 | 0.17 | 0.19 | 0.36 |
| 2013/1/16 | DL-2 | 0 | 0.27 | 0.28 | 0.55 |
| 2013/1/16 | DM-1 | 0 | 0.3 | 0.29 | 0.59 |
| 2013/1/16 | DM-2 | 0 | 0.44 | 0.33 | 0.77 |
| 2013/1/16 | DM-3 | 0 | 0.33 | 0.41 | 0.74 |
| 2013/1/16 | DH-1 | 0 | 0.34 | 0.47 | 0.81 |
| 2013/1/16 | DH-2 | 0 | 0.41 | 0.37 | 0.78 |
| 2013/1/16 | DH-3 | 0 | 0.29 | 0.37 | 0.66 |
| 2013/1/16 | EL-1 | 0.03 | 0.37 | 0.27 | 0.67 |
| 2013/1/16 | EL-2 | 0 | 0.32 | 0.29 | 0.61 |
| 2013/1/16 | EM-1 | 0 | 0.22 | 0.08 | 0.30 |
| 2013/1/16 | EM-2 | 0 | 0.4 | 0.43 | 0.83 |
| 2013/1/16 | EM-3 | 0 | 0.38 | 0.49 | 0.87 |
| 2013/1/16 | EH-1 | 0 | 0.43 | 0.44 | 0.87 |
| 2013/1/16 | EH-2 | 0 | 0.39 | 0.45 | 0.84 |
| 2013/1/16 | EH-3 | 0.07 | 0.4 | 0.28 | 0.75 |
| 2013/1/16 | FL-1 | 0 | 0.26 | 0.28 | 0.54 |
| 2013/1/16 | FL-2 | 0 | 0.31 | 0.29 | 0.60 |
| 2013/1/16 | FM-1 | 0 | 0.36 | 0.2 | 0.56 |
| 2013/1/16 | FM-2 | 0.01 | 0.66 | 0.47 | 1.14 |
| 2013/1/16 | FM-3 | 0 | 0.52 | 0.67 | 1.19 |
| 2013/1/16 | FH-1 | 0 | 0.64 | 0.48 | 1.12 |
| 2013/1/16 | FH-2 | 0.03 | 0.6 | 0.59 | 1.22 |
| 2013/1/16 | FH-3 | 0 | 0.4 | 0.59 | 0.99 |
| 2013/1/17 | AL-1 | 0 | 0.14 | 0.17 | 0.31 |
| 2013/1/17 | AL-2 | 0 | 0.14 | 0.17 | 0.31 |
| 2013/1/17 | AM-1 | 0 | 0.15 | 0.11 | 0.26 |
| 2013/1/17 | AM-2 | 0 | 0.18 | 0.24 | 0.42 |
| 2013/1/17 | AM-3 | 0.07 | 0.31 | 0.07 | 0.45 |
| 2013/1/17 | AH-1 | 0 | 0.24 | 0.1 | 0.34 |
| 2013/1/17 | AH-2 | 0 | 0.16 | 0.08 | 0.24 |
| 2013/1/17 | AH-3 | 0 | 0.13 | 0.14 | 0.27 |
| 2013/1/17 | BL-1 | 0 | 0.17 | 0.1 | 0.27 |
| 2013/1/17 | BL-2 | 0 | 0.26 | 0.19 | 0.45 |
| 2013/1/17 | BM-1 | 0 | 0.21 | 0.17 | 0.38 |
| 2013/1/17 | BM-2 | 0 | 0.32 | 0.3 | 0.62 |
| 2013/1/17 | BM-3 | 0 | 0.25 | 0.31 | 0.56 |
| 2013/1/17 | BH-1 | 0 | 0.26 | 0.29 | 0.55 |
| 2013/1/17 | BH-2 | 0 | 0.19 | 0.18 | 0.37 |
| 2013/1/17 | BH-3 | 0 | 0.28 | 0.18 | 0.46 |
| 2013/1/17 | CL-1 | 0 | 0.12 | 0.11 | 0.23 |
| 2013/1/17 | CL-2 | 0 | 0.14 | 0.23 | 0.37 |
| 2013/1/17 | CM-1 | 0 | 0.25 | 0.2 | 0.45 |
| 2013/1/17 | CM-2 | 0 | 0.35 | 0.26 | 0.61 |
| 2013/1/17 | CM-3 | 0 | 0.28 | 0.23 | 0.51 |
| 2013/1/17 | CH-1 | 0 | 0.35 | 0.35 | 0.70 |
| 2013/1/17 | CH-2 | 0 | 0.32 | 0.36 | 0.68 |
| 2013/1/17 | CH-3 | 0 | 0.32 | 0.42 | 0.74 |
| 2013/1/17 | DL-1 | 0 | 0.13 | 0.16 | 0.29 |
| 2013/1/17 | DL-2 | 0 | 0.27 | 0.27 | 0.54 |
| 2013/1/17 | DM-1 | 0 | 0.3 | 0.2 | 0.50 |
| 2013/1/17 | DM-2 | 0 | 0.34 | 0.35 | 0.69 |
| 2013/1/17 | DM-3 | 0 | 0.36 | 0.47 | 0.83 |
| 2013/1/17 | DH-1 | 0 | 0.38 | 0.52 | 0.90 |
| 2013/1/17 | DH-2 | 0 | 0.53 | 0.28 | 0.81 |
| 2013/1/17 | DH-3 | 0 | 0.36 | 0.41 | 0.77 |
| 2013/1/17 | EL-1 | 0 | 0.25 | 0.16 | 0.41 |
| 2013/1/17 | EL-2 | 0.13 | 0.32 | 0.27 | 0.72 |
| 2013/1/17 | EM-1 | 0 | 0.2 | 0.07 | 0.27 |
| 2013/1/17 | EM-2 | 0.01 | 0.58 | 0.43 | 1.02 |
| 2013/1/17 | EM-3 | 0 | 0.37 | 0.41 | 0.78 |
| 2013/1/17 | EH-1 | 0 | 0.37 | 0.34 | 0.71 |
| 2013/1/17 | EH-2 | 0 | 0.32 | 0.51 | 0.83 |
| 2013/1/17 | EH-3 | 0.07 | 0.43 | 0.17 | 0.67 |
| 2013/1/17 | FL-1 | 0 | 0.23 | 0.19 | 0.42 |
| 2013/1/17 | FL-2 | 0 | 0.36 | 0.31 | 0.67 |
| 2013/1/17 | FM-1 | 0.1 | 0.55 | 0.38 | 1.03 |
| 2013/1/17 | FM-2 | 0 | 0.53 | 0.55 | 1.08 |
| 2013/1/17 | FM-3 | 0 | 0.65 | 0.57 | 1.22 |
| 2013/1/17 | FH-1 | 0 | 0.54 | 0.86 | 1.40 |
| 2013/1/17 | FH-2 | 0 | 0.51 | 0.67 | 1.18 |
| 2013/1/17 | FH-3 | 0 | 0.5 | 0.78 | 1.28 |
| 2013/1/18 | AL-1 | 0 | 0.13 | 0.12 | 0.25 |
| 2013/1/18 | AL-2 | 0 | 0.14 | 0.13 | 0.27 |
| 2013/1/18 | AM-1 | 0 | 0.13 | 0.17 | 0.30 |
| 2013/1/18 | AM-2 | 0 | 0.16 | 0.2 | 0.36 |
| 2013/1/18 | AM-3 | 0 | 0.24 | 0.27 | 0.51 |
| 2013/1/18 | AH-1 | 0 | 0.15 | 0.19 | 0.34 |
| 2013/1/18 | AH-2 | 0 | 0.17 | 0.24 | 0.41 |
| 2013/1/18 | AH-3 | 0 | 0.18 | 0.25 | 0.43 |
| 2013/1/18 | BL-1 | 0 | 0.2 | 0.2 | 0.40 |
| 2013/1/18 | BL-2 | 0.01 | 0.32 | 0.16 | 0.49 |
| 2013/1/18 | BM-1 | 0 | 0.52 | 0.25 | 0.77 |
| 2013/1/18 | BM-2 | 0 | 0.31 | 0.45 | 0.76 |
| 2013/1/18 | BM-3 | 0 | 0.46 | 0.41 | 0.87 |
| 2013/1/18 | BH-1 | 0 | 0.47 | 0.44 | 0.91 |
| 2013/1/18 | BH-2 | 0 | 0.3 | 0.49 | 0.79 |
| 2013/1/18 | BH-3 | 0 | 0.36 | 0.44 | 0.80 |
| 2013/1/18 | CL-1 | 0 | 0.2 | 0.21 | 0.41 |
| 2013/1/18 | CL-2 | 0 | 0.26 | 0.23 | 0.49 |
| 2013/1/18 | CM-1 | 0 | 0.38 | 0.49 | 0.87 |
| 2013/1/18 | CM-2 | 0 | 0.36 | 0.55 | 0.91 |
| 2013/1/18 | CM-3 | 0 | 0.32 | 0.55 | 0.87 |
| 2013/1/18 | CH-1 | 0 | 0.39 | 0.68 | 1.07 |
| 2013/1/18 | CH-2 | 0 | 0.64 | 0.48 | 1.12 |
| 2013/1/18 | CH-3 | 0 | 0.31 | 0.58 | 0.89 |
| 2013/1/18 | DL-1 | 0 | 0.33 | 0.22 | 0.55 |
| 2013/1/18 | DL-2 | 0 | 0.23 | 0.3 | 0.53 |
| 2013/1/18 | DM-1 | 0 | 0.71 | 0.34 | 1.05 |
| 2013/1/18 | DM-2 | 0 | 0.36 | 0.62 | 0.98 |
| 2013/1/18 | DM-3 | 0 | 0.38 | 0.64 | 1.02 |
| 2013/1/18 | DH-1 | 0 | 0.37 | 0.72 | 1.09 |
| 2013/1/18 | DH-2 | 0 | 0.42 | 0.65 | 1.07 |
| 2013/1/18 | DH-3 | 0 | 0.55 | 0.57 | 1.12 |
| 2013/1/18 | EL-1 | 0 | 0.27 | 0.24 | 0.51 |
| 2013/1/18 | EL-2 | 0 | 0.29 | 0.26 | 0.55 |
| 2013/1/18 | EM-1 | 0 | 0.25 | 0.08 | 0.33 |
| 2013/1/18 | EM-2 | 0 | 0.33 | 0.43 | 0.76 |
| 2013/1/18 | EM-3 | 0 | 0.33 | 0.41 | 0.74 |
| 2013/1/18 | EH-1 | 0 | 0.39 | 0.55 | 0.94 |
| 2013/1/18 | EH-2 | 0 | 0.51 | 0.42 | 0.93 |
| 2013/1/18 | EH-3 | 0 | 0.38 | 0.45 | 0.83 |
| 2013/1/18 | FL-1 | 0 | 0.24 | 0.28 | 0.52 |
| 2013/1/18 | FL-2 | 0 | 0.31 | 0.32 | 0.63 |
| 2013/1/18 | FM-1 | 0 | 0.51 | 1.29 | 1.80 |
| 2013/1/18 | FM-2 | 0 | 0.79 | 1.09 | 1.88 |
| 2013/1/18 | FM-3 | 0 | 0.64 | 1.56 | 2.20 |
| 2013/1/18 | FH-1 | 0 | 1.19 | 1.41 | 2.60 |
| 2013/1/18 | FH-2 | 0 | 0.52 | 1.39 | 1.91 |
| 2013/1/18 | FH-3 | 0 | 0.79 | 1.62 | 2.41 |
| 2013/1/19 | AL-1 | 0 | 0.12 | 0.18 | 0.30 |
| 2013/1/19 | AL-2 | 0 | 0.14 | 0.19 | 0.33 |
| 2013/1/19 | AM-1 | 0 | 0.22 | 0.35 | 0.57 |
| 2013/1/19 | AM-2 | 0 | 0.26 | 0.38 | 0.64 |
| 2013/1/19 | AM-3 | 0 | 0.28 | 0.34 | 0.62 |
| 2013/1/19 | AH-1 | 0 | 0.25 | 0.37 | 0.62 |
| 2013/1/19 | AH-2 | 0 | 0.58 | 0.2 | 0.78 |
| 2013/1/19 | AH-3 | 0 | 0.21 | 0.39 | 0.60 |
| 2013/1/19 | BL-1 | 0 | 0.13 | 0.19 | 0.32 |
| 2013/1/19 | BL-2 | 0 | 0.32 | 0.19 | 0.51 |
| 2013/1/19 | BM-1 | 0 | 0.59 | 0.49 | 1.08 |
| 2013/1/19 | BM-2 | 0 | 0.53 | 0.65 | 1.18 |
| 2013/1/19 | BM-3 | 0 | 0.3 | 0.71 | 1.01 |
| 2013/1/19 | BH-1 | 0 | 0.43 | 0.66 | 1.09 |
| 2013/1/19 | BH-2 | 0 | 0.64 | 0.65 | 1.29 |
| 2013/1/19 | BH-3 | 0 | 0.36 | 0.71 | 1.07 |
| 2013/1/19 | CL-1 | 0.06 | 0.34 | 0.19 | 0.59 |
| 2013/1/19 | CL-2 | 0.07 | 0.35 | 0.21 | 0.63 |
| 2013/1/19 | CM-1 | 0 | 0.31 | 0.79 | 1.10 |
| 2013/1/19 | CM-2 | 0 | 0.41 | 0.97 | 1.38 |
| 2013/1/19 | CM-3 | 0 | 0.35 | 0.87 | 1.22 |
| 2013/1/19 | CH-1 | 0 | 0.78 | 0.89 | 1.67 |
| 2013/1/19 | CH-2 | 0 | 0.34 | 1.08 | 1.42 |
| 2013/1/19 | CH-3 | 0 | 0.35 | 1.02 | 1.37 |
| 2013/1/19 | DL-1 | 0 | 0.26 | 0.25 | 0.51 |
| 2013/1/19 | DL-2 | 0 | 0.24 | 0.3 | 0.54 |
| 2013/1/19 | DM-1 | 0 | 0.45 | 1.04 | 1.49 |
| 2013/1/19 | DM-2 | 0 | 0.86 | 1.17 | 2.03 |
| 2013/1/19 | DM-3 | 0 | 0.54 | 1.39 | 1.93 |
| 2013/1/19 | DH-1 | 0 | 0.36 | 1.43 | 1.79 |
| 2013/1/19 | DH-2 | 0 | 0.45 | 1.41 | 1.86 |
| 2013/1/19 | DH-3 | 0 | 0.35 | 1.31 | 1.66 |
| 2013/1/19 | EL-1 | 0 | 0.17 | 0.31 | 0.48 |
| 2013/1/19 | EL-2 | 0 | 0.19 | 0.33 | 0.52 |
| 2013/1/19 | EM-1 | 0 | 0.54 | 0.21 | 0.75 |
| 2013/1/19 | EM-2 | 0 | 0.4 | 1.15 | 1.55 |
| 2013/1/19 | EM-3 | 0 | 0.59 | 1.25 | 1.84 |
| 2013/1/19 | EH-1 | 0 | 0.82 | 1.05 | 1.87 |
| 2013/1/19 | EH-2 | 0 | 0.24 | 1.39 | 1.63 |
| 2013/1/19 | EH-3 | 0 | 0.69 | 1.04 | 1.73 |
| 2013/1/19 | FL-1 | 0 | 0.38 | 0.33 | 0.71 |
| 2013/1/19 | FL-2 | 0 | 0.27 | 0.35 | 0.62 |
| 2013/1/19 | FM-1 | 0 | 0.82 | 3.85 | 4.67 |
| 2013/1/19 | FM-2 | 0 | 0.47 | 3.64 | 4.11 |
| 2013/1/19 | FM-3 | 0 | 0.58 | 4.36 | 4.94 |
| 2013/1/19 | FH-1 | 0 | 0.88 | 4.23 | 5.11 |
| 2013/1/19 | FH-2 | 0 | 0.36 | 4.89 | 5.25 |
| 2013/1/19 | FH-3 | 0 | 0.56 | 4.89 | 5.45 |
| 2013/1/20 | AL-1 | 0 | 0.12 | 0.17 | 0.29 |
| 2013/1/20 | AL-2 | 0 | 0.16 | 0.17 | 0.33 |
| 2013/1/20 | AM-1 | 0 | 0.31 | 0.57 | 0.88 |
| 2013/1/20 | AM-2 | 0 | 0.35 | 0.67 | 1.02 |
| 2013/1/20 | AM-3 | 0 | 0.28 | 0.69 | 0.97 |
| 2013/1/20 | AH-1 | 0 | 0.31 | 0.63 | 0.94 |
| 2013/1/20 | AH-2 | 0 | 0.28 | 0.61 | 0.89 |
| 2013/1/20 | AH-3 | 0 | 0.27 | 0.69 | 0.96 |
| 2013/1/20 | BL-1 | 0 | 0.3 | 0.15 | 0.45 |
| 2013/1/20 | BL-2 | 0 | 0.17 | 0.24 | 0.41 |
| 2013/1/20 | BM-1 | 0 | 0.31 | 1.22 | 1.53 |
| 2013/1/20 | BM-2 | 0 | 0.49 | 1.59 | 2.08 |
| 2013/1/20 | BM-3 | 0 | 0.36 | 1.48 | 1.84 |
| 2013/1/20 | BH-1 | 0 | 0.36 | 1.37 | 1.73 |
| 2013/1/20 | BH-2 | 0 | 0.37 | 1.4 | 1.77 |
| 2013/1/20 | BH-3 | 0 | 0.34 | 1.32 | 1.66 |
| 2013/1/20 | CL-1 | 0 | 0.15 | 0.19 | 0.34 |
| 2013/1/20 | CL-2 | 0 | 0.19 | 0.27 | 0.46 |
| 2013/1/20 | CM-1 | 0 | 0.28 | 2.12 | 2.40 |
| 2013/1/20 | CM-2 | 0 | 0 | 2.63 | 2.63 |
| 2013/1/20 | CM-3 | 0 | 0 | 2.37 | 2.37 |
| 2013/1/20 | CH-1 | 0 | 0.39 | 2.3 | 2.69 |
| 2013/1/20 | CH-2 | 0 | 0 | 2.66 | 2.66 |
| 2013/1/20 | CH-3 | 0 | 0 | 2.32 | 2.32 |
| 2013/1/20 | DL-1 | 0 | 0.24 | 0.23 | 0.47 |
| 2013/1/20 | DL-2 | 0 | 0.24 | 0.26 | 0.50 |
| 2013/1/20 | DM-1 | 0 | 0 | 2.77 | 2.77 |
| 2013/1/20 | DM-2 | 0 | 0 | 3.21 | 3.21 |
| 2013/1/20 | DM-3 | 0 | 0 | 4.12 | 4.12 |
| 2013/1/20 | DH-1 | 0 | 0 | 3.67 | 3.67 |
| 2013/1/20 | DH-2 | 0 | 0 | 3.87 | 3.87 |
| 2013/1/20 | DH-3 | 0 | 0 | 3.6 | 3.60 |
| 2013/1/20 | EL-1 | 0 | 0.35 | 0.34 | 0.69 |
| 2013/1/20 | EL-2 | 0 | 0.25 | 0.36 | 0.61 |
| 2013/1/20 | EM-1 | 0 | 0.61 | 0.18 | 0.79 |
| 2013/1/20 | EM-2 | 0 | 0.2 | 2.79 | 2.99 |
| 2013/1/20 | EM-3 | 0 | 0 | 3.62 | 3.62 |
| 2013/1/20 | EH-1 | 0 | 0.33 | 3.07 | 3.40 |
| 2013/1/20 | EH-2 | 0 | 0 | 2.77 | 2.77 |
| 2013/1/20 | EH-3 | 0 | 0 | 3.08 | 3.08 |
| 2013/1/20 | FL-1 | 0 | 0.4 | 0.38 | 0.78 |
| 2013/1/20 | FL-2 | 0 | 0.32 | 0.46 | 0.78 |
| 2013/1/20 | FM-1 | 0 | 0 | 10.87 | 10.87 |
| 2013/1/20 | FM-2 | 0 | 0 | 9.92 | 9.92 |
| 2013/1/20 | FM-3 | 0 | 0 | 11.14 | 11.14 |
| 2013/1/20 | FH-1 | 0 | 0 | 9.92 | 9.92 |
| 2013/1/20 | FH-2 | 0 | 0 | 10.53 | 10.53 |
| 2013/1/20 | FH-3 | 0 | 0 | 11.35 | 11.35 |
| 2013/1/21 | AL-1 | 0 | 0.09 | 0.26 | 0.35 |
| 2013/1/21 | AL-2 | 0 | 0.14 | 0.22 | 0.36 |
| 2013/1/21 | AM-1 | 0 | 0.42 | 0.8 | 1.22 |
| 2013/1/21 | AM-2 | 0 | 0.32 | 1.05 | 1.37 |
| 2013/1/21 | AM-3 | 0 | 0.27 | 1.14 | 1.41 |
| 2013/1/21 | AH-1 | 0 | 0.37 | 0.95 | 1.32 |
| 2013/1/21 | AH-2 | 0 | 0.41 | 0.82 | 1.23 |
| 2013/1/21 | AH-3 | 0 | 0.37 | 0.94 | 1.31 |
| 2013/1/21 | BL-1 | 0 | 0.22 | 0.31 | 0.53 |
| 2013/1/21 | BL-2 | 0 | 0.2 | 0.33 | 0.53 |
| 2013/1/21 | BM-1 | 0 | 0.44 | 1.54 | 1.98 |
| 2013/1/21 | BM-2 | 0 | 0.38 | 2.37 | 2.75 |
| 2013/1/21 | BM-3 | 0 | 0 | 2.36 | 2.36 |
| 2013/1/21 | BH-1 | 0 | 0.4 | 1.64 | 2.04 |
| 2013/1/21 | BH-2 | 0 | 0.42 | 1.97 | 2.39 |
| 2013/1/21 | BH-3 | 0 | 0.61 | 1.71 | 2.32 |
| 2013/1/21 | CL-1 | 0 | 0.29 | 0.43 | 0.72 |
| 2013/1/21 | CL-2 | 0 | 0.33 | 0.39 | 0.72 |
| 2013/1/21 | CM-1 | 0 | 0.15 | 2.48 | 2.63 |
| 2013/1/21 | CM-2 | 0 | 0 | 3.91 | 3.91 |
| 2013/1/21 | CM-3 | 0 | 0.68 | 3.77 | 4.45 |
| 2013/1/21 | CH-1 | 0 | 0 | 4 | 4.00 |
| 2013/1/21 | CH-2 | 0 | 0.34 | 4.05 | 4.39 |
| 2013/1/21 | CH-3 | 0 | 0 | 3.48 | 3.48 |
| 2013/1/21 | DL-1 | 0 | 0.24 | 0.43 | 0.67 |
| 2013/1/21 | DL-2 | 0 | 0.28 | 0.4 | 0.68 |
| 2013/1/21 | DM-1 | 0 | 0.16 | 3.12 | 3.28 |
| 2013/1/21 | DM-2 | 0 | 0 | 4.65 | 4.65 |
| 2013/1/21 | DM-3 | 0 | 0 | 5.15 | 5.15 |
| 2013/1/21 | DH-1 | 0 | 0 | 4.92 | 4.92 |
| 2013/1/21 | DH-2 | 0 | 0 | 4.28 | 4.28 |
| 2013/1/21 | DH-3 | 0 | 0 | 4.27 | 4.27 |
| 2013/1/21 | EL-1 | 0 | 0.26 | 0.48 | 0.74 |
| 2013/1/21 | EL-2 | 0 | 0.34 | 0.63 | 0.97 |
| 2013/1/21 | EM-1 | 0 | 0.67 | 0.31 | 0.98 |
| 2013/1/21 | EM-2 | 0 | 0.26 | 2.65 | 2.91 |
| 2013/1/21 | EM-3 | 0 | 0.21 | 3.98 | 4.19 |
| 2013/1/21 | EH-1 | 0 | 0.04 | 3.69 | 3.73 |
| 2013/1/21 | EH-2 | 0 | 0 | 2.86 | 2.86 |
| 2013/1/21 | EH-3 | 0 | 0.14 | 3.02 | 3.16 |
| 2013/1/21 | FL-1 | 0 | 0.42 | 0.58 | 1.00 |
| 2013/1/21 | FL-2 | 0 | 0.62 | 0.57 | 1.19 |
| 2013/1/21 | FM-1 | 0 | 0 | 11.65 | 11.65 |
| 2013/1/21 | FM-2 | 0 | 0 | 10.72 | 10.72 |
| 2013/1/21 | FM-3 | 0.13 | 0 | 11.75 | 11.88 |
| 2013/1/21 | FH-1 | 0 | 0 | 10.89 | 10.89 |
| 2013/1/21 | FH-2 | 0 | 0 | 11.45 | 11.45 |
| 2013/1/21 | FH-3 | 0 | 0 | 11.61 | 11.61 |
| 2013/1/22 | AL-1 | 0 | 0.16 | 0.17 | 0.33 |
| 2013/1/22 | AL-2 | 0 | 0.16 | 0.27 | 0.43 |
| 2013/1/22 | AM-1 | 0 | 0.25 | 0.83 | 1.08 |
| 2013/1/22 | AM-2 | 0 | 0.48 | 0.93 | 1.41 |
| 2013/1/22 | AM-3 | 0 | 0.43 | 0.96 | 1.39 |
| 2013/1/22 | AH-1 | 0 | 0.33 | 0.96 | 1.29 |
| 2013/1/22 | AH-2 | 0 | 0.33 | 0.92 | 1.25 |
| 2013/1/22 | AH-3 | 0 | 0.31 | 0.95 | 1.26 |
| 2013/1/22 | BL-1 | 0 | 0.19 | 0.34 | 0.53 |
| 2013/1/22 | BL-2 | 0 | 0.15 | 0.37 | 0.52 |
| 2013/1/22 | BM-1 | 0 | 0.25 | 1.8 | 2.05 |
| 2013/1/22 | BM-2 | 0 | 0.46 | 2.41 | 2.87 |
| 2013/1/22 | BM-3 | 0 | 0.8 | 2.13 | 2.93 |
| 2013/1/22 | BH-1 | 0 | 0.32 | 2.06 | 2.38 |
| 2013/1/22 | BH-2 | 0 | 0 | 2.54 | 2.54 |
| 2013/1/22 | BH-3 | 0 | 0.29 | 1.96 | 2.25 |
| 2013/1/22 | CL-1 | 0 | 0.21 | 0.33 | 0.54 |
| 2013/1/22 | CL-2 | 0 | 0.29 | 0.39 | 0.68 |
| 2013/1/22 | CM-1 | 0 | 0 | 2.9 | 2.90 |
| 2013/1/22 | CM-2 | 0 | 0 | 4.09 | 4.09 |
| 2013/1/22 | CM-3 | 0 | 0 | 4.12 | 4.12 |
| 2013/1/22 | CH-1 | 0 | 0 | 4.32 | 4.32 |
| 2013/1/22 | CH-2 | 0 | 0 | 3.99 | 3.99 |
| 2013/1/22 | CH-3 | 0 | 0 | 3.7 | 3.70 |
| 2013/1/22 | DL-1 | 0 | 0.22 | 0.47 | 0.69 |
| 2013/1/22 | DL-2 | 0 | 0.24 | 0.46 | 0.70 |
| 2013/1/22 | DM-1 | 0 | 0.37 | 2.87 | 3.24 |
| 2013/1/22 | DM-2 | 0 | 0.07 | 4.58 | 4.65 |
| 2013/1/22 | DM-3 | 0 | 0 | 6.17 | 6.17 |
| 2013/1/22 | DH-1 | 0 | 0.14 | 4.85 | 4.99 |
| 2013/1/22 | DH-2 | 0 | 0 | 4.97 | 4.97 |
| 2013/1/22 | DH-3 | 0 | 0 | 4.48 | 4.48 |
| 2013/1/22 | EL-1 | 0 | 0.28 | 0.52 | 0.80 |
| 2013/1/22 | EL-2 | 0 | 0.23 | 0.56 | 0.79 |
| 2013/1/22 | EM-1 | 0 | 0.73 | 0.38 | 1.11 |
| 2013/1/22 | EM-2 | 0 | 0.47 | 2.96 | 3.43 |
| 2013/1/22 | EM-3 | 0 | 0 | 4.59 | 4.59 |
| 2013/1/22 | EH-1 | 0 | 0.06 | 4.29 | 4.35 |
| 2013/1/22 | EH-2 | 0 | 0.21 | 3.4 | 3.61 |
| 2013/1/22 | EH-3 | 0 | 0.16 | 3.81 | 3.97 |
| 2013/1/22 | FL-1 | 0 | 0.67 | 0.59 | 1.26 |
| 2013/1/22 | FL-2 | 0 | 0.58 | 0.79 | 1.37 |
| 2013/1/22 | FM-1 | 0 | 0 | 11.18 | 11.18 |
| 2013/1/22 | FM-2 | 0 | 0 | 11.82 | 11.82 |
| 2013/1/22 | FM-3 | 0.26 | 0 | 11.09 | 11.35 |
| 2013/1/22 | FH-1 | 0 | 0 | 12.48 | 12.48 |
| 2013/1/22 | FH-2 | 0 | 0 | 13.52 | 13.52 |
| 2013/1/22 | FH-3 | 0 | 0 | 12.82 | 12.82 |
| 2013/1/23 | AL-1 | 0 | 0.07 | 0.07 | 0.14 |
| 2013/1/23 | AL-2 | 0 | 0.19 | 0.17 | 0.36 |
| 2013/1/23 | AM-1 | 0 | 0.32 | 0.78 | 1.10 |
| 2013/1/23 | AM-2 | 0 | 0.35 | 1.11 | 1.46 |
| 2013/1/23 | AM-3 | 0 | 0.37 | 0.91 | 1.28 |
| 2013/1/23 | AH-1 | 0 | 0.43 | 0.99 | 1.42 |
| 2013/1/23 | AH-2 | 0 | 0.32 | 0.97 | 1.29 |
| 2013/1/23 | AH-3 | 0 | 0.35 | 1.08 | 1.43 |
| 2013/1/23 | BL-1 | 0 | 0.18 | 0.33 | 0.51 |
| 2013/1/23 | BL-2 | 0 | 0.22 | 0.36 | 0.58 |
| 2013/1/23 | BM-1 | 0 | 0.44 | 1.68 | 2.12 |
| 2013/1/23 | BM-2 | 0 | 0.29 | 3.05 | 3.34 |
| 2013/1/23 | BM-3 | 0 | 0.2 | 2.26 | 2.46 |
| 2013/1/23 | BH-1 | 0.02 | 0.36 | 0.23 | 0.61 |
| 2013/1/23 | BH-2 | 0 | 0.2 | 0.37 | 0.57 |
| 2013/1/23 | BH-3 | 0 | 0.3 | 1.84 | 2.14 |
| 2013/1/23 | CL-1 | 0 | 0.28 | 2.36 | 2.64 |
| 2013/1/23 | CL-2 | 0 | 0.67 | 1.84 | 2.51 |
| 2013/1/23 | CM-1 | 0 | 0 | 2.87 | 2.87 |
| 2013/1/23 | CM-2 | 0 | 0 | 4.11 | 4.11 |
| 2013/1/23 | CM-3 | 0 | 0.17 | 4.63 | 4.80 |
| 2013/1/23 | CH-1 | 0 | 0.21 | 4.13 | 4.34 |
| 2013/1/23 | CH-2 | 0 | 0 | 4.11 | 4.11 |
| 2013/1/23 | CH-3 | 0 | 0 | 3.73 | 3.73 |
| 2013/1/23 | DL-1 | 0 | 0.33 | 0.41 | 0.74 |
| 2013/1/23 | DL-2 | 0 | 0.31 | 0.44 | 0.75 |
| 2013/1/23 | DM-1 | 0 | 0 | 3.16 | 3.16 |
| 2013/1/23 | DM-2 | 0 | 0 | 4.62 | 4.62 |
| 2013/1/23 | DM-3 | 0 | 0 | 6.69 | 6.69 |
| 2013/1/23 | DH-1 | 0 | 0 | 5.34 | 5.34 |
| 2013/1/23 | DH-2 | 0 | 0 | 4.86 | 4.86 |
| 2013/1/23 | DH-3 | 0 | 0 | 4.21 | 4.21 |
| 2013/1/23 | EL-1 | 0 | 0.36 | 0.32 | 0.68 |
| 2013/1/23 | EL-2 | 0.06 | 0.35 | 0.38 | 0.79 |
| 2013/1/23 | EM-1 | 0 | 0.67 | 0.34 | 1.01 |
| 2013/1/23 | EM-2 | 0 | 0.51 | 2.06 | 2.57 |
| 2013/1/23 | EM-3 | 0 | 0.15 | 3.88 | 4.03 |
| 2013/1/23 | EH-1 | 0.09 | 0.25 | 3.32 | 3.66 |
| 2013/1/23 | EH-2 | 0 | 0.35 | 2.24 | 2.59 |
| 2013/1/23 | EH-3 | 0 | 0.38 | 2.57 | 2.95 |
| 2013/1/23 | FL-1 | 0 | 0.47 | 0.32 | 0.79 |
| 2013/1/23 | FL-2 | 0.07 | 0.47 | 0.52 | 1.06 |
| 2013/1/23 | FM-1 | 0.66 | 0 | 5.53 | 6.19 |
| 2013/1/23 | FM-2 | 0.56 | 0 | 6.32 | 6.88 |
| 2013/1/23 | FM-3 | 0.51 | 0.68 | 4.23 | 5.42 |
| 2013/1/23 | FH-1 | 0.75 | 0 | 7.84 | 8.59 |
| 2013/1/23 | FH-2 | 0.68 | 0 | 9.13 | 9.81 |
| 2013/1/23 | FH-3 | 0.58 | 0 | 4.23 | 4.81 |
| 2013/1/24 | AL-1 | 0 | 0.12 | 0.2 | 0.32 |
| 2013/1/24 | AL-2 | 0 | 0.16 | 0.16 | 0.32 |
| 2013/1/24 | AM-1 | 0 | 0.29 | 0.67 | 0.96 |
| 2013/1/24 | AM-2 | 0 | 0.37 | 0.78 | 1.15 |
| 2013/1/24 | AM-3 | 0 | 0.42 | 1.16 | 1.58 |
| 2013/1/24 | AH-1 | 0.23 | 0.6 | 1.26 | 2.09 |
| 2013/1/24 | AH-2 | 0 | 0.78 | 1.37 | 2.15 |
| 2013/1/24 | AH-3 | 0 | 0.43 | 1.12 | 1.55 |
| 2013/1/24 | BL-1 | 0 | 0.22 | 0.45 | 0.67 |
| 2013/1/24 | BL-2 | 0 | 0.19 | 0.47 | 0.66 |
| 2013/1/24 | BM-1 | 0 | 0.32 | 1.72 | 2.04 |
| 2013/1/24 | BM-2 | 0 | 0.66 | 4.18 | 4.84 |
| 2013/1/24 | BM-3 | 0 | 0.48 | 2.67 | 3.15 |
| 2013/1/24 | BH-1 | 0 | 0.39 | 2.23 | 2.62 |
| 2013/1/24 | BH-2 | 0 | 0 | 6.83 | 6.83 |
| 2013/1/24 | BH-3 | 0 | 0.35 | 2.27 | 2.62 |
| 2013/1/24 | CL-1 | 0 | 0.25 | 0.21 | 0.46 |
| 2013/1/24 | CL-2 | 0 | 0.31 | 0.18 | 0.49 |
| 2013/1/24 | CM-1 | 0 | 0.33 | 2.31 | 2.64 |
| 2013/1/24 | CM-2 | 0 | 0.41 | 2.35 | 2.76 |
| 2013/1/24 | CM-3 | 0 | 0.51 | 2.46 | 2.97 |
| 2013/1/24 | CH-1 | 0 | 0.51 | 2.76 | 3.27 |
| 2013/1/24 | CH-2 | 0 | 0 | 5.58 | 5.58 |
| 2013/1/24 | CH-3 | 0 | 0.54 | 3.56 | 4.10 |
| 2013/1/24 | DL-1 | 0 | 0.28 | 0.42 | 0.70 |
| 2013/1/24 | DL-2 | 0 | 0.27 | 0.49 | 0.76 |
| 2013/1/24 | DM-1 | 0 | 0 | 3.3 | 3.30 |
| 2013/1/24 | DM-2 | 0 | 0 | 5.79 | 5.79 |
| 2013/1/24 | DM-3 | 0 | 0.32 | 5.54 | 5.86 |
| 2013/1/24 | DH-1 | 0.19 | 0 | 4.91 | 5.10 |
| 2013/1/24 | DH-2 | 0.11 | 0.19 | 7.9 | 8.20 |
| 2013/1/24 | DH-3 | 0 | 0 | 5.38 | 5.38 |
| 2013/1/24 | EL-1 | 0 | 0.41 | 0.48 | 0.89 |
| 2013/1/24 | EL-2 | 0 | 0.35 | 0.69 | 1.04 |
| 2013/1/24 | EM-1 | 0 | 0.83 | 0.47 | 1.30 |
| 2013/1/24 | EM-2 | 0 | 0.27 | 4.06 | 4.33 |
| 2013/1/24 | EM-3 | 0 | 0.23 | 4.86 | 5.09 |
| 2013/1/24 | EH-1 | 0.11 | 0.06 | 4.87 | 5.04 |
| 2013/1/24 | EH-2 | 0 | 0.35 | 4.26 | 4.61 |
| 2013/1/24 | EH-3 | 0 | 0.09 | 4.07 | 4.16 |
| 2013/1/24 | FL-1 | 0 | 0.46 | 0.22 | 0.68 |
| 2013/1/24 | FL-2 | 0.09 | 0.33 | 0.46 | 0.88 |
| 2013/1/24 | FM-1 | 0.41 | 0.62 | 2.67 | 3.70 |
| 2013/1/24 | FM-2 | 0.83 | 0.7 | 4.07 | 5.60 |
| 2013/1/24 | FM-3 | 0.2 | 1.57 | 1.34 | 3.11 |
| 2013/1/24 | FH-1 | 0.8 | 0 | 4.28 | 5.08 |
| 2013/1/24 | FH-2 | 1.65 | 0 | 7.96 | 9.61 |
| 2013/1/24 | FH-3 | 0.58 | 0.43 | 2.12 | 3.13 |
| 2013/1/25 | AL-1 | 0.06 | 0.29 | 0.26 | 0.61 |
| 2013/1/25 | AL-2 | 0 | 0.13 | 0.17 | 0.30 |
| 2013/1/25 | AM-1 | 0 | 0.31 | 1.01 | 1.32 |
| 2013/1/25 | AM-2 | 0 | 0.56 | 1.24 | 1.80 |
| 2013/1/25 | AM-3 | 0 | 0.32 | 1.39 | 1.71 |
| 2013/1/25 | AH-1 | 0 | 0.57 | 1.09 | 1.66 |
| 2013/1/25 | AH-2 | 0 | 0.5 | 1.27 | 1.77 |
| 2013/1/25 | AH-3 | 0 | 0.43 | 1.6 | 2.03 |
| 2013/1/25 | BL-1 | 0 | 0.28 | 0.36 | 0.64 |
| 2013/1/25 | BL-2 | 0 | 0.24 | 0.49 | 0.73 |
| 2013/1/25 | BM-1 | 0 | 0.49 | 2.83 | 3.32 |
| 2013/1/25 | BM-2 | 0 | 0.69 | 3.93 | 4.62 |
| 2013/1/25 | BM-3 | 0 | 0.41 | 3.47 | 3.88 |
| 2013/1/25 | BH-1 | 0 | 0.7 | 3.48 | 4.18 |
| 2013/1/25 | BH-2 | 0 | 0.58 | 3.81 | 4.39 |
| 2013/1/25 | BH-3 | 0 | 0.43 | 3.1 | 3.53 |
| 2013/1/25 | CL-1 | 0 | 0.19 | 0.28 | 0.47 |
| 2013/1/25 | CL-2 | 0.41 | 0.24 | 0.72 | 1.37 |
| 2013/1/25 | CM-1 | 0 | 0 | 3.91 | 3.91 |
| 2013/1/25 | CM-2 | 0 | 0 | 7.43 | 7.43 |
| 2013/1/25 | CM-3 | 0 | 0.4 | 5.09 | 5.49 |
| 2013/1/25 | CH-1 | 0.31 | 0 | 7.72 | 8.03 |
| 2013/1/25 | CH-2 | 0 | 0 | 7.92 | 7.92 |
| 2013/1/25 | CH-3 | 0 | 0 | 4.55 | 4.55 |
| 2013/1/25 | DL-1 | 0.06 | 0.38 | 0.3 | 0.74 |
| 2013/1/25 | DL-2 | 0 | 0.24 | 0.54 | 0.78 |
| 2013/1/25 | DM-1 | 0 | 0.25 | 3.91 | 4.16 |
| 2013/1/25 | DM-2 | 0 | 1.02 | 5.48 | 6.50 |
| 2013/1/25 | DM-3 | 0.44 | 0 | 6.41 | 6.85 |
| 2013/1/25 | DH-1 | 0.29 | 0 | 5.77 | 6.06 |
| 2013/1/25 | DH-2 | 0.38 | 0 | 6.18 | 6.56 |
| 2013/1/25 | DH-3 | 0.13 | 0 | 7.65 | 7.78 |
| 2013/1/25 | EL-1 | 0 | 0.63 | 0.63 | 1.26 |
| 2013/1/25 | EL-2 | 0 | 0.39 | 0.52 | 0.91 |
| 2013/1/25 | EM-1 | 0 | 0.99 | 0.5 | 1.49 |
| 2013/1/25 | EM-2 | 0.42 | 0 | 6.22 | 6.64 |
| 2013/1/25 | EM-3 | 0.1 | 0.21 | 4.8 | 5.11 |
| 2013/1/25 | EH-1 | 0.2 | 0 | 3.25 | 3.45 |
| 2013/1/25 | EH-2 | 0.28 | 0.2 | 2.67 | 3.15 |
| 2013/1/25 | EH-3 | 0.2 | 0 | 6.53 | 6.73 |
| 2013/1/25 | FL-1 | 0.13 | 0.43 | 0.25 | 0.81 |
| 2013/1/25 | FL-2 | 0.26 | 0.24 | 0.69 | 1.19 |
| 2013/1/25 | FM-1 | 0.52 | 1.49 | 1.69 | 3.70 |
| 2013/1/25 | FM-2 | 0.54 | 1.29 | 2.22 | 4.05 |
| 2013/1/25 | FM-3 | 1.62 | 5.19 | 4.84 | 11.65 |
| 2013/1/25 | FH-1 | 0 | 1.68 | 1.01 | 2.69 |
| 2013/1/25 | FH-2 | 0.85 | 1.72 | 2.04 | 4.61 |
| 2013/1/25 | FH-3 | 1.44 | 1.91 | 2.97 | 6.32 |
